# Gut microbiota-mediated biotransformation of dietary bioactive compounds: inter-individual variability, determinants, and implications for personalised nutrition

**DOI:** 10.3389/fsysb.2026.1898890

**Published:** 2026-08-07

**Authors:** Tapash Chakraborty, Shashanka Jyoti Kashyap, Pragyan Deep Chetia, Sumona Purkayastha

**Affiliations:** School of Pharmaceutical Sciences, Girijananda Chowdhury University, Guwahati, Assam, India

**Keywords:** dietary bioactives, fermented foods, gut microbiota, inter-individual variability, personalised nutrition, polyphenol biotransformation, probiotic

## Abstract

Dietary bioactive compounds, particularly polyphenols and glucosinolates, are widely associated with reduced risk of chronic disease, yet fewer than 10% of most ingested polyphenols reach systemic circulation in their active form, a figure that varies markedly by compound class; resveratrol bioavailability is below 1%, whereas some simple phenolic acids may reach 30%–50%. The gut microbiota is the primary site where these compounds are chemically transformed into absorbable metabolites, but individuals vary enormously in their capacity to carry out this transformation. This review examines how gut microbiota-mediated biotransformation of dietary bioactive compounds is determined by three interacting factors: baseline microbiota composition, fermented food consumption, and probiotic or prebiotic supplementation. The review covers polyphenols, isoflavones, ellagitannins, glucosinolates, and alkaloids. Synthetic drugs and non-dietary administration routes are excluded. The three-factor framework collectively determines an individual’s biotransformation phenotype, which governs how much of a given dietary bioactive reaches the bloodstream as an active metabolite. Documented examples include equol production from daidzein (present in only around 30% of Western adults), urolithin generation from ellagitannins (three distinct metabotypes in the population), and dihydroberberine production from berberine. Each of these variations produces measurable differences in health outcomes, including cardiovascular markers, glycaemic control, and muscle function. Biotransformation phenotyping is technically feasible through urine metabolomics and faecal enzyme assays. Personalised dietary guidance that accounts for an individual’s biotransformation capacity has the potential to resolve the inconsistency that has characterised polyphenol intervention trials. Standardised phenotyping protocols, re-analysis of existing trial datasets, and updated regulatory frameworks represent the most urgent research priorities.

## 1 Introduction

Dietary bioactive compounds, particularly polyphenols and glucosinolates, are secondary plant metabolites that epidemiological studies consistently associate with lower rates of cardiovascular disease, type 2 diabetes, and certain cancers (Rocchetti et al., 2022). Translating this epidemiological signal into reliable clinical benefits has proved difficult, and the central reason is bioavailability. Fewer than 10% of ingested polyphenols reach systemic circulation in their active, native form, although this proportion is compound-class dependent: aglycone-poor, highly conjugated molecules such as resveratrol fall below 1% bioavailability, while structurally simpler phenolic acids may reach 30%–50% (Hu et al., 2024). The remaining majority pass through the small intestine unabsorbed and arrive in the colon, where they encounter the enzymatic machinery of the gut microbiota (Hu et al., 2024).

The gut microbiota transforms these compounds through a range of reactions including deglycosylation, reduction, ring fission, and esterase cleavage. The resulting smaller molecules are absorbed from the colon and enter circulation as the metabolites that produce measurable physiological effects (Seyed Hameed et al., 2020). This means that the health benefit from a polyphenol-rich meal is not determined solely by what a person eats. It is also determined by the composition and enzymatic capacity of the microbial community in their colon.

Clinical trials of dietary polyphenols have historically treated the gut microbiota as a background variable, recruiting mixed populations and averaging their responses. This approach has generated contradictory results across studies, with some reporting clear benefits and others finding none. The explanation, in many cases, is that the trials mixed producers and non-producers of the relevant microbial metabolites without distinguishing between them (Espín et al., 2024).

This review organises the determinants of individual biotransformation capacity into three factors. These three were selected because each acts directly on the colonic enzymatic repertoire available to process dietary bioactives, and because each is independently modifiable through diet or supplementation, which gives the framework direct translational relevance; other variables that influence the gut microbiota more broadly, such as medication use, smoking, physical activity, and circadian rhythm, are acknowledged determinants of overall microbiota composition but are not addressed here because their evidence base specific to dietary bioactive biotransformation, as opposed to microbiota composition in general, remains comparatively sparse. The first factor is baseline microbiota composition, which is shaped by host genetics, age, geographic origin, and long-term dietary habits. This baseline determines the pool of enzymes available for processing dietary bioactives and is resistant to short-term change (Rengasamy, 2026). The second factor is fermented food consumption. Fermented foods alter the biotransformation equation in two ways: they deliver compounds that have already been partially transformed before ingestion, and they introduce live microorganisms that can transiently modify the resident microbiota (Cevallos-Fernández et al., 2026). The third factor is targeted probiotic and prebiotic supplementation, which can enrich specific biotransforming bacterial populations or introduce organisms with defined enzymatic capabilities (Shahid, 2025).

Together, these three factors determine what this review calls the biotransformation phenotype of an individual: the net capacity of a person’s gut microbiota to convert parent dietary compounds into bioavailable, biologically active metabolites. Understanding this phenotype provides the mechanistic basis for personalized dietary recommendations.

Reviews on polyphenol bioavailability, fermented food health effects, and probiotic supplementation have largely treated these as separate topics. The integration of all three into a unified framework, with inter-individual variability as the central organising concept and personalised nutrition as the translational output, has not been systematically addressed. This review provides that integration.

**FIGURE 1:**
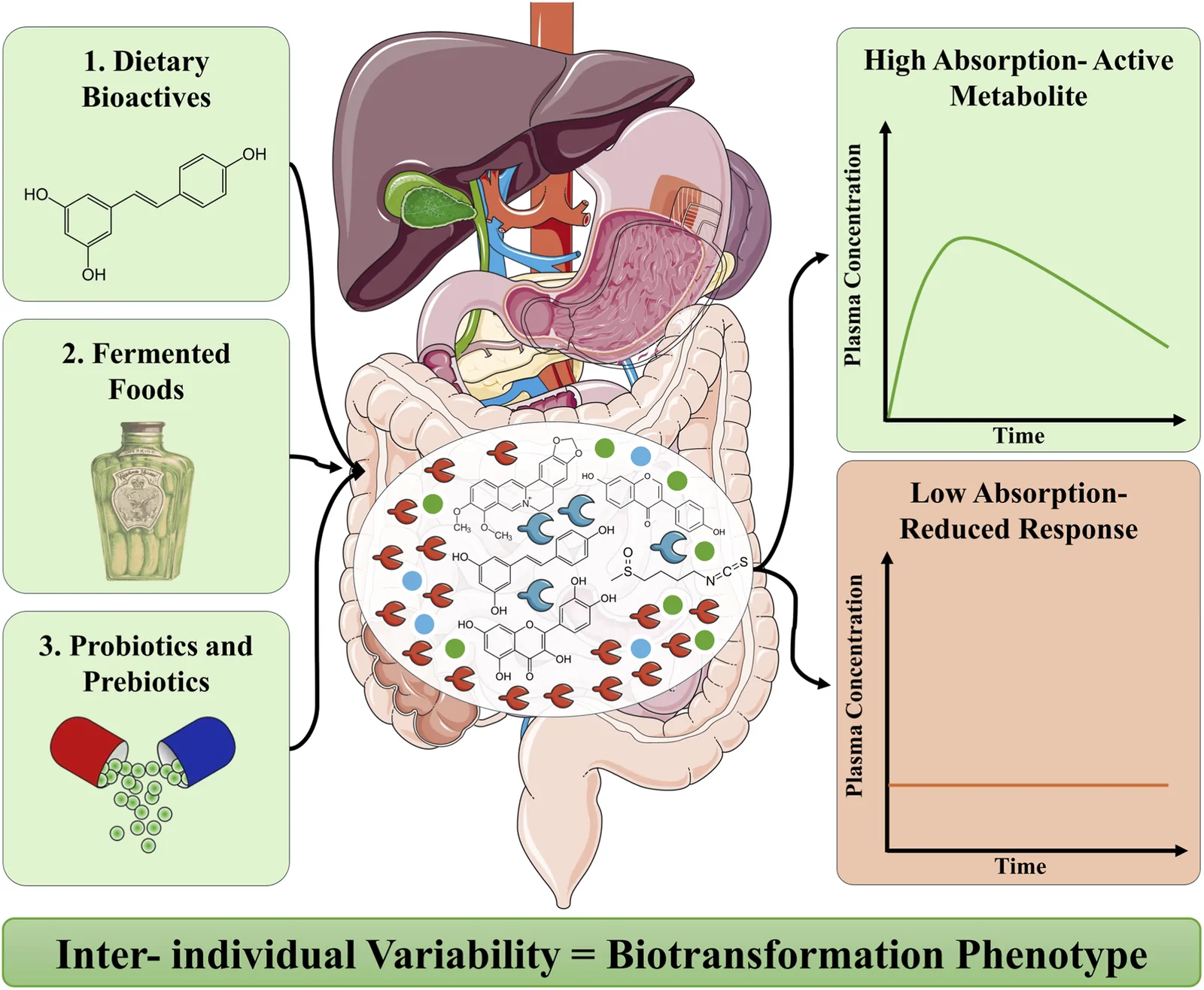
Graphical abstract summarising the three-factor framework of the review. Dietary bioactive intake, fermented food consumption, and probiotic or prebiotic supplementation converge on the gut microbial community, which performs enzymatic biotransformation reactions. Individuals with high biotransformation capacity generate abundant circulating metabolites and measurable health benefits; those with low capacity excrete parent compounds largely unchanged. Inter-individual divergence in health outcomes from identical dietary intake is therefore a function of biotransformation phenotype. Abbreviations: DHB, dihydroberberine; EQ, equol; UA, urolithin A.

The scope covers dietary bioactive compounds consumed through food, including flavonoids, phenolic acids, stilbenes, ellagitannins, isoflavones, glucosinolates, and alkaloids such as berberine, with oral bioavailability as the primary measured outcome. The biotransformation mechanisms examined are microbial deglycosylation, reduction, ring fission, and deconjugation in both the *ex vivo* fermentation and *in vivo* gut contexts. Pharmaceutical-route administration of these or related compounds (intravenous, topical, or as isolated synthetic agents) falls outside this scope. The three-factor framework and its downstream consequences for biotransformation phenotype and health outcome are summarised in Figure 1.

## 2 Dietary bioactive compounds and their bioavailability challenges

### 2.1 Major classes and structural barriers

Flavonoids are the most abundant class of dietary polyphenols and exist in plant foods primarily as glycosides, with one or more sugar units attached to a phenolic backbone (Fernandes et al., 2023). The molecular weights of monomeric flavonoids range from roughly 270–600 Da; polymeric forms such as proanthocyanidins and condensed tannins fall well outside this range and can exceed 1,500 Da and are treated separately below under ellagitannins. The glycoside form is largely unable to cross the intestinal epithelium by passive diffusion because the attached sugars increase hydrophilicity and molecular size beyond what passive transport can accommodate (Ferreira et al., 2026).

Phenolic acids are simpler in structure but often physically trapped within the plant cell wall, esterified to structural polysaccharides such as cellulose and pectin. This physical entrapment limits their release during digestion in the upper gastrointestinal tract and restricts early absorption (Fernandes et al., 2023), although food processing, cooking, fermentation, and mechanical disruption such as milling or juicing, can mechanically or enzymatically free a substantial fraction of these bound phenolics before the food is even consumed, a point developed further in Section 4. Stilbenes, of which resveratrol is the most studied, illustrate a different problem: even when absorbed in the small intestine, they undergo rapid phase II conjugation in the enterocytes and liver, and the resulting glucuronide and sulphate conjugates are pharmacologically inactive. The net result is that free, unconjugated resveratrol is rarely detected at meaningful concentrations in human plasma after dietary consumption (Seyed Hameed et al., 2020); the microbial metabolites that partly compensate for this rapid clearance are discussed in Section 2.3.

Ellagitannins are high-molecular-weight hydrolysable tannins, often exceeding 1,500 Da, found in pomegranates, walnuts, and berries. They are not absorbed intact from the gastrointestinal tract at any stage. To exert systemic effects, they must first be hydrolysed in the colon to ellagic acid, which is then sequentially modified by specific gut bacteria into smaller lactone compounds called urolithins (e.g., urolithin A, urolithin B, and isourolithin A) (Wojciechowska and Kujawska, 2023).

Glucosinolates require a two-step activation. Plant-derived myrosinase released during chewing begins the conversion to isothiocyanates such as sulforaphane. However, much of this conversion depends on microbial thioglucosidases in the colon, particularly when plant myrosinase is inactivated during cooking (Cevallos-Fernández et al., 2026). The alkaloid berberine faces a distinct barrier: intestinal epithelial P-glycoprotein pumps actively transport it back into the gut lumen, limiting its net absorption. Microbial reduction to dihydroberberine (DHB) changes the molecule’s interaction with these efflux pumps and allows substantially more to enter circulation (Seyed Hameed et al., 2020); the specific bacterial reductases responsible are described in Section 3.1.

### 2.2 The colon as the primary transformation site

As noted above, the great majority of ingested polyphenols reach the colon unabsorbed. The protective food matrix surrounding these compounds helps them survive the acidic conditions of the stomach, but the same physical embedding restricts access by small intestinal digestive enzymes (Ferreira et al., 2026). The few simple phenolic compounds that are absorbed in the jejunum are quickly glucuronidated, sulphated, or methylated, and most of these conjugates are exported through the bile back into the duodenum.

This biliary excretion sets up a process called enterohepatic recycling. Phase II conjugates travel down the intestinal tract to the colon, where microbial beta-glucuronidases and sulfatases remove the conjugating groups, restoring the free aglycone or metabolite, which can then be reabsorbed. This cycle extends the residence time of bioactive metabolites in the body substantially beyond what would be expected from a single absorption event (Deng et al., 2026).

The human genome encodes a restricted set of digestive enzymes that are not equipped to break the range of glycosidic, ester, and polymeric bonds present in dietary plant matrices. The colonic microbiota fills this gap with an enzymatic repertoire of considerable breadth. The chemical transformation that occurs in the colon is not an incidental metabolic event. It is the primary mechanism through which most dietary bioactives become biologically available (Alharbi, 2026).

### 2.3 Key compound-specific examples

Daidzein, the soy isoflavone, undergoes microbial reduction in the colon to produce equol. Individuals who harbour the relevant bacteria achieve plasma equol concentrations roughly 3 to 5 times higher than non-producers following the same dietary isoflavone intake (rather than total isoflavone metabolites generally), along with measurably different cardiovascular and hormonal effects (Espín et al., 2024). About 30% of adults in Western populations produce equol, compared to over 50% in Asian populations with a tradition of fermented soy consumption (Espín et al., 2024).

Ellagitannins from pomegranates and walnuts are converted by gut bacteria first to ellagic acid and then to a series of urolithin metabolites. Three distinct population phenotypes have been defined based on which urolithins appear in urine: metabotype A produces urolithin A, metabotype B produces a mixture including urolithin B and isourolithin A, and metabotype 0 produces no urolithins at all (Wojciechowska and Kujawska, 2023; Zelenović et al., 2026). Urolithin A and isourolithin A are generated via distinct bacterial pathways, the former chiefly through Gordonibacter species and the latter through Ellagibacter isourolithinifaciens (detailed in Section 3.2), so metabotype B reflects a partial or alternative enzymatic route rather than simply an incomplete version of metabotype A. These phenotypes are fixed by the microbial composition of the individual and determine entirely whether ellagitannin-rich foods provide systemic anti-inflammatory and mitophagy-promoting benefits.

Berberine is reductively modified by colonic bacteria to dihydroberberine. DHB is not recognised as a substrate by P-glycoprotein efflux pumps, and its intestinal absorption is roughly five times greater than that of the parent compound, a figure established in pharmacokinetic comparisons reviewed by Seyed Hameed et al. (2020) and consistent with the mechanistic data reported by Li et al. (2022). After absorption, host tissues re-oxidise DHB back to berberine, which then exerts its pharmacological effects on metabolic pathways (Seyed Hameed et al., 2020; Li et al., 2022).

Resveratrol illustrates the importance of microbial metabolism for sustained systemic presence. Following its rapid phase II clearance (Section 2.1), colonic bacteria generate dihydroresveratrol and related metabolites that enter circulation and are detected in plasma and urine for considerably longer than the parent compound (Seyed Hameed et al., 2020). Anthocyanins undergo ring fission in the colon to produce simple phenolic acids, most commonly including protocatechuic acid and vanillic acid among a wider range of ring-fission products, which are the species that reach plasma at measurable concentrations and exert anti-inflammatory activity (Di Pede et al., 2023). The structural barriers, microbial enzyme classes, and biotransformation outcomes for all major dietary bioactive compound classes covered in this review are summarised in Table 1.

**TABLE 1:** Major dietary bioactive compound classes, their primary structural barriers to intestinal absorption, the gut microbial enzymatic reactions that produce bioavailable metabolites, and the resulting absorption or bioactivity outcomes.

Compound class	Key representative compound	Primary structural barrier	Primary microbial transformation	Key enzyme(s)	Biotransformed product(s)	Absorption/activity outcome	References
Flavonols	Quercetin	Complex glycosylation	Deglycosylation	β-Glucosidases	Quercetin aglycone	Enhanced bioaccessibility and local colonic antioxidant capacity	Hu et al. (2024)
Flavonols	Rutin	Bulky O-rutinose attachment	C-ring cleavage	Ring-fission dioxygenases	3,4-Dihydroxyphenylacetic acid	Systemic circulation enhancement	Alharbi (2026)
Flavanones	Hesperidin	Rutinoside conjugation	Hydrolysis	α-L-Rhamnosidases	Hesperetin	Enhanced mucosal transport	Hu et al. (2024)
Flavanones	Naringin	Glycosylation	O-glycosidic cleavage	β-Glucosidases	Naringenin	Increased lipophilicity for transepithelial diffusion	Cevallos-Fernández et al. (2026)
Isoflavones	Daidzein	Glycoside form (Daidzin)	Sequential reduction	Daidzein reductase	Equol	Superior estrogenic receptor affinity and antioxidant capacity	Renukadevi et al. (2026)
Isoflavones	Genistein	Glycoside form (Genistin)	C-ring cleavage	Ring-fission dioxygenases	O-desmethylangolensin	Biotransformation phenotype-dependent physiological efficacy	Seyed Hameed et al. (2020)
Anthocyanins	Malvidin	Glycosylation and pH lability	Deglycosylation and fission	β-Glucosidases	Syringic acid	Systemic anti-inflammatory effects	Rocchetti et al. (2022)
Anthocyanins	Cyanidin	Complex pigmentation	Ring cleavage	Ring-fission dioxygenases	Protocatechuic acid	Bioavailable secondary metabolite circulation	Di Pede et al. (2023)
Flavan-3-ols	Epigallocatechin gallate	Gallated esterification	Ester hydrolysis	Microbial tannases	Epigallocatechin, gallic acid	Modulated lipid metabolism and antioxidant retention	Ferreira et al. (2026)
Flavan-3-ols	Procyanidin B2	Oligomeric condensation	Depolymerization	C–C bond cleavers	Phenyl-γ-valerolactones	Sustained systemic antioxidant currency	Di Pede et al. (2023)
Flavan-3-ols	Catechin	Stereochemical constraints	Dehydroxylation and cleavage	Microbial reductases	Phenylpropionic acids	Phenylpropionic acids detected in plasma at micromolar concentrations, associated with reduced oxidative stress markers	Ferreira et al. (2026)
Phenolic acids	Chlorogenic acid	Esterified to quinic acid	De-esterification	Cinnamoyl esterases	Caffeic acid, quinic acid	Rapid transepithelial diffusion	Cevallos-Fernández et al. (2026)
Phenolic acids	Ferulic acid	Physical entrapment in cell wall	Decarboxylation	Phenolic-acid decarboxylases	4-Vinylguaiacol	Increased mucosal transport kinetics	Rocchetti et al. (2022)
Stilbenes	trans-Resveratrol	Rapid host Phase II conjugation	Aliphatic double-bond reduction	NADH-dependent reductases	Dihydroresveratrol	Prolonged cardiometabolic protection	Espín et al. (2024)
Stilbenes	Pterostilbene	Phase II clearance	Demethylation	Microbial demethylases	Pinostilbene	Phenotype-specific systemic bioavailability	Alharbi (2026)
Ellagitannins	Punicalagin	High molecular weight (>1,500 Da)	Hydrolysis	Microbial tannases	Ellagic acid	Liberates ellagic acid, which is then further metabolised by gut bacteria to urolithins	Ferreira et al. (2026)
Ellagitannins	Ellagic acid	Low aqueous solubility	Lactone-ring cleavage, dehydroxylation	Microbial dehydroxylases	Urolithin A	Mitophagy activation and cardioprotective phenotypes	García-Villalba et al. (2022)
Glucosinolates	Glucoraphanin	Inert precursor structure	Dual-transformation hydrolysis	Microbial thioglucosidases	Sulforaphane	Nrf2 antioxidant pathway activation	Cevallos-Fernández et al. (2026)
Alkaloids	Berberine	Apical P-glycoprotein efflux	Reductive modification	Bacterial reductases	Dihydroberberine	Efflux evasion yielding elevated maximal plasma concentration	Seyed Hameed et al. (2020)

Compound classes are arranged in order of structural complexity. Two entries (Catechin; Pterostilbene) and one Ellagitannin entry (Punicalagin) have been revised per reviewer comment to remove vague or misleading wording. Abbreviations: GH, glycoside hydrolase; NADH, nicotinamide adenine dinucleotide (reduced); O-DMA, O-desmethylangolensin; SCFA, short-chain fatty acid.

## 3 Gut microbiota as the biotransformation engine

### 3.1 The enzymatic repertoire

The colonic microbiota carries out dietary bioactive biotransformation through several defined enzyme classes. Glycoside hydrolases, particularly beta-glucosidases from glycoside hydrolase (GH) families 1 and 3, and beta-glucuronidases from GH family 79 according to the carbohydrate-active enzyme (CAZy) classification, are responsible for cleaving sugar units from glycosylated compounds (Peng et al., 2022). This deglycosylation step is usually a prerequisite for absorption, since the resulting aglycone is smaller, more lipophilic, and more membrane-permeable than the parent glycoside.

NADH- and NADPH-dependent reductases carry out the reduction of double bonds and carbonyl groups under the anaerobic conditions of the colon. Several of these enzymes, including daidzein reductase, are oxygen-sensitive and lose activity on exposure to even brief aerobic conditions, which has practical implications for *ex vivo* fermentation assays (Section 7.2): sample handling and incubation must preserve strict anaerobiosis or biotransformation capacity will be systematically underestimated. These reductive reactions modify the electronic structure of target molecules and, in the case of berberine and daidzein, alter their interaction with efflux transporters and their biological activity (Seyed Hameed et al., 2020).

Ring-fission enzymes, including the largely anaerobic dioxygenases specific to ellagic acid and related substrates, cleave aromatic rings to generate simpler phenolic acid derivatives that are rapidly absorbed from the colonic epithelium (Tow et al., 2026).

Microbial thioglucosidases convert glucosinolates to isothiocyanates in the colon, supplementing or substituting for plant-derived myrosinase depending on food preparation methods (Cevallos-Fernández et al., 2026). Lactic acid bacteria contribute decarboxylases and demethylases that modify phenolic acids, though these activities are not universal across the group. Certain strains of Lactiplantibacillus plantarum, for example, produce gallate decarboxylase, converting gallic acid derivatives into smaller, more readily absorbed products (Shakya et al., 2021).

Beta-glucuronidases also play a direct role in enterohepatic recycling by removing glucuronic acid from phase II conjugates exported through bile, regenerating free aglycones for reabsorption (Deng et al., 2026). This activity is a double-edged sword: the same deconjugating capacity that regenerates bioactive aglycones for reabsorption also regenerates potentially toxic compounds, including certain drug metabolites and procarcinogens, that the host had specifically conjugated for excretion, a tension that complicates any simple framing of beta-glucuronidase activity as uniformly beneficial.

### 3.2 Key biotransforming taxa

Equol production from daidzein depends entirely on bacteria within the family Coriobacteriaceae. Slackia isoflavoniconvertens and Slackia equolifaciens are the principal species responsible for the daidzein-to-equol reduction cascade (Gangwar et al., 2026), although the complete pathway proceeds through multiple enzymatic intermediates, and some of these intermediate steps may be supplied by other community members through cross-feeding rather than by Slackia alone (see Section 5.1 for the practical implications of this for probiotic colonisation). These organisms are not universally present. Their prevalence in the colon is shaped by long-term dietary exposure to soy, which is why equol production rates differ between Asian and Western populations (Espín et al., 2024).

Urolithin production from ellagitannins requires the sequential activity of at least two specialised bacterial species, both members of the family Eggerthellaceae. Gordonibacter urolithinfaciens catalyses the early dehydroxylation steps that convert ellagic acid to intermediate urolithins, while Ellagibacter isourolithinifaciens carries out the terminal dehydroxylation that produces isourolithin A (Wojciechowska and Kujawska, 2023). The presence or absence of these organisms in an individual’s colon determines their urolithin metabotype.

*Lactobacillus* and *Bifidobacterium* species are broad-spectrum deglycosylation agents, using their esterase and glycoside hydrolase inventories to release aglycones from a wide range of flavonoid glycosides (Peng et al., 2022). *Bacteroides* and *Prevotella* species initiate ring fission of flavonoids, generating phenolic acid intermediates that downstream bacteria can further metabolise (Hu et al., 2024).

*Clostridium* and *Eubacterium* species specialize in the anaerobic reduction of flavonoids and stilbenes under the strict anaerobic conditions of the distal colon. Within this group, *Eubacterium* ramulus is notable specifically as a key organism for flavonoid C-ring cleavage rather than reduction alone, generating ring-opened phenolic acid products in addition to its reductive activity (Seyed Hameed et al., 2020).

*Faecalibacterium prausnitzii* does not directly biotransform polyphenols, but its butyrate production maintains the integrity of the colonic epithelium and the transport conditions for absorbing microbial metabolites (Xu et al., 2024). Butyrate also contributes to maintaining low epithelial oxygen tension in the colon by supporting colonocyte oxidative metabolism, and this oxygen-poor environment is itself a precondition for the obligately anaerobic reductive and ring-fission reactions described above, linking *F. prausnitzii* indirectly to the broader biotransformation network even though it does not perform these reactions itself.

### 3.3 Factors that shape biotransformation capacity

Long-term dietary pattern is the strongest determinant of which biotransforming bacteria colonise the colon. Sustained consumption of diverse plant foods selects for the specific bacterial guilds that metabolise them. Conversely, diets low in plant fibre and polyphenols do not support these populations, and their biotransformation capacity declines accordingly (Rengasamy, 2026). This is a stable, long-term effect rather than a rapid response to acute dietary change.

Geography and cultural dietary traditions have created population-level differences in biotransformation phenotypes. The higher prevalence of Slackia species in Asian populations relative to Western populations reflects multigenerational habitual soy consumption. This is a microbiota-diet interaction rather than a genetic effect: individuals from Asian backgrounds raised on Western diets show equol production rates like Western populations, a finding specifically reported within the broader coevolutionary analysis of Espín et al. (2024) (Espín et al., 2024).

Age reduces biotransformation capacity through a progressive decline in *Bifidobacterium* abundance and a general contraction of microbial diversity (Rudzka et al., 2025). Broad-spectrum antibiotic use disrupts the anaerobic communities responsible for reductive biotransformation, although the degree of disruption and the time to recovery depend substantially on antibiotic class and duration: agents with greater activity against anaerobes, such as clindamycin, tend to produce more prolonged disruption of reductive taxa than agents such as amoxicillin with a narrower anaerobic spectrum. Studies examining equol and urolithin production before and after antibiotic courses show substantial reductions that can persist for months (Rengasamy, 2026).

**FIGURE 2:**
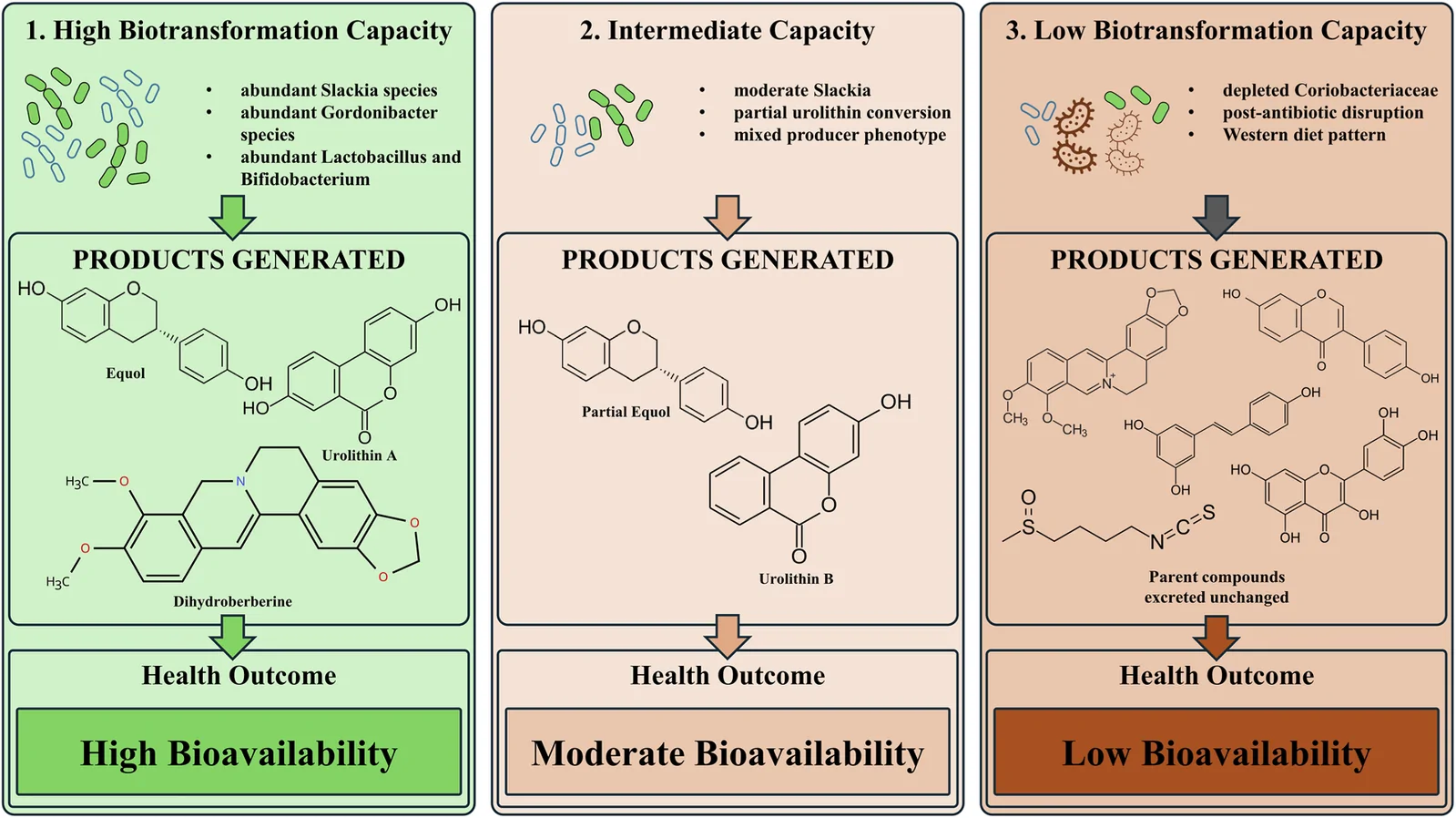
Schematic representation of the gut microbiota biotransformation capacity spectrum. Left panel (high capacity): diverse colon enriched with *Slackia*, *Gordonibacter*, *Lactobacillus*, and *Bifidobacterium* species, producing equol, urolithin A, and dihydroberberine from their precursors. Centre panel (intermediate capacity): partial producer phenotypes yielding urolithin B and inconsistent equol output. Right panel (low capacity): depleted microbiota with absent specialist taxa and low systemic metabolite exposure. Bioavailability index bars below each panel indicate relative systemic metabolite levels. Abbreviations: DHB, dihydroberberine; EQ, equol; UA, urolithin A.

Host immune genetics indirectly influence microbiota composition through mucosal immune selection, contributing to the highly individual nature of the colonic microbial community. Variation in pattern-recognition and antigen-presentation genes, including NOD2 and HLA loci, has been linked to differences in mucosal immune tone that shape which bacterial taxa are tolerated and which are selectively constrained, providing one plausible mechanistic route by which host genetics could influence biotransformation capacity indirectly (Shahid, 2025). The principal biotransforming taxa, their enzyme complements, and the dietary and supplementation factors that support their colonic abundance are catalogued in Table 2. The relationship between microbiota composition and biotransformation capacity across the population spectrum is illustrated in Figure 2.

**TABLE 2:** Key gut microbial taxa involved in dietary bioactive biotransformation, with their principal enzyme classes, substrate specificities, biotransformation products, and the dietary or supplementation factors that selectively enrich each taxon in the human colon.

Gut microbial taxon	Phylum	Key biotransformation enzyme	Substrate compound class	Biotransformation product	Factors increasing abundance	References
*Slackia isoflavoniconvertens*	Actinomycetota	Daidzein reductase	Isoflavones	Equol	Habitual fermented soy consumption	Seyed Hameed et al. (2020)
*Slackia equolifaciens*	Actinomycetota	Daidzein reductase	Isoflavones	Equol	Daidzein prebiotics, isoflavone-rich diets	Seyed Hameed et al. (2020)
*Gordonibacter urolithinfaciens*	Actinomycetota	Microbial dehydroxylase	Ellagitannins	Urolithin M5, Urolithin C	Pomegranate extracts, walnut consumption	Espín et al. (2024)
*Ellagibacter isourolithinifaciens*	Actinomycetota	Microbial dehydroxylase	Ellagitannins	Isourolithin A	Ellagitannin-rich fruit diets	Hu et al. (2024)
*Lactiplantibacillus plantarum*	Bacillota	β-glucosidase	Flavonoid glycosides	Flavonoid aglycones	Fermented plant matrices, kefir	Shakya et al. (2021)
*Lactobacillus acidophilus*	Bacillota	β-glucosidase	Plant glucosides	Bioaccessible aglycones	Fermented dairy products; prebiotic supplementation (specific prebiotic substrate not detailed in the primary source)	Li et al. (2022)
*Lactiplantibacillus plantarum* WCFS1	Bacillota	Gallate decarboxylase	Phenolic acids	Pyrogallol	Plant-based, gallotannin-rich polyphenol diets (e.g., grape and pomegranate polyphenols)	Shakya et al. (2021)
*Bifidobacterium adolescentis*	Actinomycetota	β-glucosidase	Complex flavonoid glycosides	Flavonoid aglycones	Inulin, FOS	Peng et al. (2022)
*Bifidobacterium pseudocatenulatum*	Actinomycetota	β-glucuronidase	Phase II conjugated polyphenols	Deconjugated aglycones	GOS, high-fibre diets	Peng et al. (2022)
*Bacteroides ovatus*	Bacteroidota	β-glucosidase	Complex plant glycosides	Bioaccessible aglycones	Plant-based dietary fibre	Hu et al. (2024)
*Bacteroides fragilis*	Bacteroidota	β-glucuronidase	Phase II conjugated polyphenols	Deconjugated secondary metabolites	High-fibre diets	Deng et al. (2026)
*Bacteroides thetaiotaomicron*	Bacteroidota	α-L-rhamnosidase	Flavanone rutinosides	Hesperetin aglycone	Complex plant carbohydrates, resistant starch	Hu et al. (2024)
*Eubacterium ramulus*	Bacillota	Ring-fission dioxygenase	Flavonols, isoflavones	Simple phenolic acids, O-DMA	Sustained polyphenol intake	Seyed Hameed et al. (2020)
*Clostridium orbiscindens*	Bacillota	C-ring cleaving dioxygenase	Flavonoids	3,4-Dihydroxyphenylacetic acid	Diverse dietary fibre and polyphenols	Hu et al. (2024)
*Ruminococcus gnavus*	Bacillota	β-glucuronidase	Conjugated bile acids, polyphenols	Deconjugated secondary metabolites	Fermentable dietary fibre	Deng et al. (2026)
*Faecalibacterium prausnitzii*	Bacillota	Butyryl-CoA: acetate-CoA-transferase	Fermentable fibres	Butyrate	Fructooligosaccharides (FOS), resistant starch	Xu et al. (2024)

*Taxa are grouped by phylum. Where the original supporting evidence specified a general dietary category, the entry has been refined here to name the specific dietary substrate identified in that evidence, per reviewer comment. The Lactobacillus acidophilus entry has additionally been qualified per reviewer comment, since the primary source does not specify the prebiotic substrate used. Abbreviations: EC, enzyme commission number; FOS, fructooligosaccharides; GH, glycoside hydrolase; GOS, galactooligosaccharides; KEGG, Kyoto Encyclopedia of Genes and Genomes.*

## 4 Fermented food consumption as a microbiota modulator

### 4.1 Upstream pre-processing: what changes before the gut

Food fermentation modifies the chemical profile of bioactive compounds before they are ingested. This is a distinct process from gut microbial biotransformation, but it acts in the same direction, and the two effects are additive. The key distinction is that food fermentation occurs outside the body and is controlled by the production process, whereas gut microbial biotransformation varies with the individual (Cevallos-Fernández et al., 2026).

In the fermentation of soybeans into natto, tempeh, and miso, microbial beta-glucosidases from the fermenting organisms hydrolyse the major isoflavone glycosides daidzin and genistin into their corresponding aglycones daidzein and genistein. Fermented soy products therefore deliver a higher ratio of aglycone to glycoside than unfermented soy, partly bypassing the intestinal deglycosylation requirement before the product even reaches the colon (West et al., 2025); the practical benefit of this bypass depends on how readily the liberated aglycone itself is absorbed, since daidzein and genistein are markedly better absorbed than their glycosides but the extent of the improvement still varies between individuals and between the two aglycones.

Fermentation of Panax ginseng drives the sequential conversion of high-molecular-weight ginsenosides such as Rb1 into minor ginsenosides including Compound K and Rg3. Fungal and bacterial hydrolases from the fermenting organisms carry out these conversions during the production process (Zhao et al., 2021). The improved bioavailability of these minor ginsenosides is driven primarily by increased lipophilicity rather than smaller molecular weight alone: Compound K, for instance, retains a single sugar moiety but its overall structure is substantially less polar than Rb1, and it is this reduced polarity that chiefly accounts for its greater membrane permeability.

In kombucha, the symbiotic community of acetic acid bacteria and yeasts oxidises tea catechins and galloylated derivatives into complex pigment polymers called theabrownins, producing a different polyphenol profile from the fresh tea used as the starting material (Ecklu-Mensah et al., 2024). Theabrownins are themselves poorly absorbed, but emerging evidence suggests they may act locally within the colon as prebiotic-like substrates that influence microbial composition, representing a distinct mechanism of action from the catechins they are derived from rather than a simple loss of bioactivity.

Lactic acid fermentation of cabbage into kimchi and sauerkraut involves glucosinolate hydrolysis by microbial thioglucosidases during fermentation, generating isothiocyanates in the food product itself before consumption. This conversion does not depend on plant myrosinase, which is often inactivated during food preparation (Cevallos-Fernández et al., 2026). The extent of this conversion is not fixed: it varies with fermentation time and temperature, and individual glucosinolates differ in how readily they undergo microbial hydrolysis, so the isothiocyanate yield of a given batch of kimchi or sauerkraut is not uniform. The practical consequence is that fermented cruciferous vegetables may deliver active isothiocyanates more reliably than their cooked unfermented equivalents, regardless of the consumer’s gut microbial composition, though the magnitude of this advantage depends on production conditions.

### 4.2 How fermented foods reshape the gut microbiota

Beyond delivering pre-transformed compounds, fermented foods introduce live microorganisms and fermentation-derived metabolites such as organic acids and bacteriocins that modify the colonic environment. These changes favour certain commensal populations and can transiently alter the biotransformation capacity of the resident community (Hamsho et al., 2026).

Regular kefir consumption has been associated with increased *Lactobacillus* and *Bifidobacterium* abundance in the colon, which corresponds to improved deglycosylation capacity for subsequently ingested polyphenol glycosides (Gupta et al., 2024; Neves et al., 2026). It should be noted that the Gupta et al. (2024) trial was conducted in critically ill adults, a population with substantially altered baseline microbiota and gut physiology; whether the same magnitude of effect holds in healthy adults consuming kefir as part of an otherwise normal diet has not been established with the same rigour (Gupta et al., 2024).

Kimchi delivers lactic acid bacteria alongside fermentable dietary fibre, and this combination has been linked to expansion of Lachnospiraceae and Ruminococcaceae (Fraiz et al., 2024; Schropp et al., 2025). These are taxonomically broad families with diverse metabolic functions: while many constituent genera are net butyrate producers, others within the same families are associated primarily with hydrogen metabolism or other fermentation end-products, so an expansion at the family level does not by itself specify which downstream metabolic function has increased.

A dietary intervention at Stanford University showed that a diet rich in diverse fermented foods over 10 weeks increased overall microbial diversity and reduced inflammatory markers in healthy adults, establishing a clear connection between fermented food intake and functional changes in the colonic ecosystem (Wastyk et al., 2021). This trial used an intake of approximately six servings of fermented foods per day, considerably higher than typical fermented food consumption in most populations, so the generalisability of the magnitude of effect to more modest, real-world intake levels remain to be established.

By contrast, a crossover trial of sauerkraut found compositional changes limited to single bacterial species following fresh and pasteurised sauerkraut consumption, with effects on the overall community structure that the authors themselves characterised as modest (Schropp et al., 2025). This finding deserves emphasis rather than treatment as a minor caveat: it indicates that the effects of fermented foods on the resident gut microbiota are frequently transient, dose-dependent, and may fail to reach statistical significance at the community level even when fermentation-derived compounds are clearly being delivered. This reflects a broader limitation in the field: the gut microbiota of healthy adults shows resilience to short-term dietary interventions, and compositional changes are easier to detect after sustained, higher-dose exposure such as that used by Wastyk et al. (2021) than after the shorter or lower-dose exposures more typical of habitual consumption (Wastyk et al., 2021).

An important caveat applies to interpreting these studies. Most investigations use 16S rRNA gene sequencing to track changes in microbial relative abundance and then infer changes in biotransformation capacity from these taxonomic data. Increased *Bifidobacterium* abundance does not guarantee increased beta-glucosidase activity. The same caution extends, to a lesser degree, to shotgun metagenomic functional gene abundance: detecting a gene is also only a proxy for its expression, not direct evidence of it (see Section 7.1). Connecting compositional or genetic shifts to actual metabolite generation requires paired metabolomics data from biological fluids, which most current fermented food studies do not provide (Shahid, 2025).

### 4.3 Health benefits associated with fermented food consumption

Beyond their role in modulating biotransformation capacity, fermented foods have an independent evidence base linking habitual consumption to broader health outcomes, which provides useful context for the mechanistic discussion above. Fermented food intake has been associated with improved markers of immune regulation, metabolic homeostasis, and reduced systemic inflammation across clinical and preclinical studies, with effects attributed jointly to the live microorganisms delivered, fermentation-derived bioactive metabolites, and downstream shifts in resident microbial ecology (Chen et al., 2019; Wastyk et al., 2021; Spencer et al., 2022). Reported associations also extend to cognitive function, consistent with a wider gut–brain signalling role for fermentation-derived metabolites, although the strength of evidence varies considerably by health domain and by the specific fermented food studied. Notably, inter-individual variability in response to fermented foods is itself a recurring theme in this literature, reinforcing the case made throughout this review that fermented food consumption should be considered alongside, rather than as a substitute for, individual biotransformation phenotyping when designing personalised dietary strategies (Tillisch et al., 2013; Zmora et al., 2018; Gu et al., 2020).

### 4.4 Fermented food-derived bioactives as direct substrates

Some fermented foods deliver bioactive compounds that exert effects directly, without requiring further microbial transformation in the colon. Lactic acid fermentation of dairy proteins generates bioactive peptides, including ACE-inhibitory sequences that can be absorbed through the small intestinal epithelium and contribute to blood pressure reduction (Cevallos-Fernández et al., 2026). Not all such peptides survive intact to the point of absorption: many ACE-inhibitory sequences are degraded by gastrointestinal proteases, and the peptides that do reach the small intestinal epithelium in active form are typically short, stable di- or tripeptides rather than the full range of peptides generated during fermentation.

GABA is produced during the fermentation of kimchi, soy products, and yogurt by Lactobacillus-mediated glutamate decarboxylation. Pre-formed GABA in fermented foods provides direct substrate for intestinal receptors and enters the enteric nervous system without depending on *de novo* microbial synthesis in the colon (Cevallos-Fernández et al., 2026); because GABA does not cross the blood–brain barrier in significant quantities, its physiological effects from dietary sources are understood to be primarily peripheral, acting through the gut–brain axis rather than directly on the central nervous system. Some fermentation strains synthesise high-molecular-weight exopolysaccharides from simple sugars. These fermentation-derived polysaccharides have been proposed to function as prebiotics in the colon, selectively enriching the bacteria responsible for their own synthesis, although the evidence specific to exopolysaccharide self-enrichment loops is preliminary and the supporting reference addresses prebiotic effects more generally rather than this mechanism specifically (Holmes et al., 2022).

**FIGURE 3:**
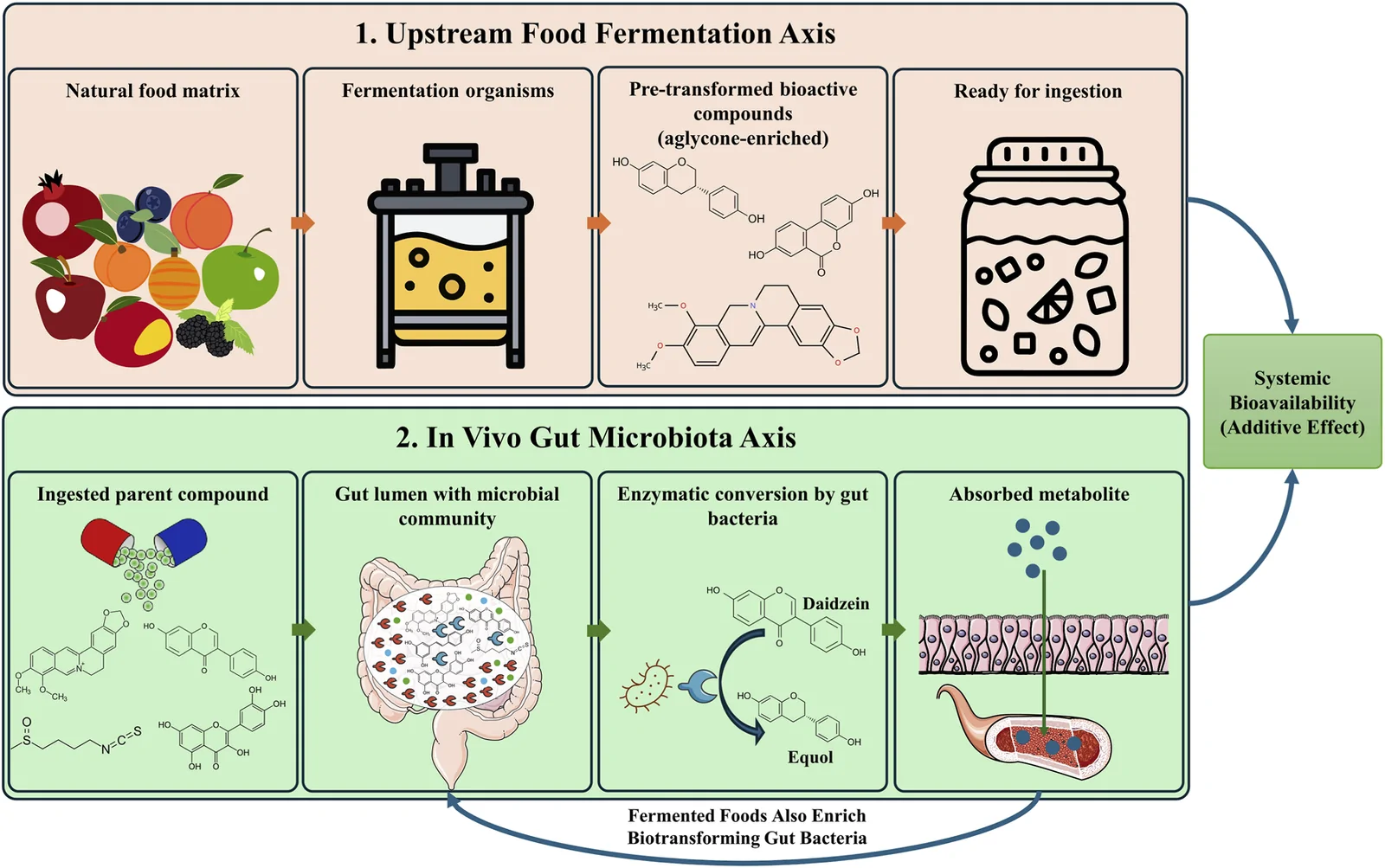
Dual-axis diagram illustrating two parallel and additive biotransformation pathways. The upper track represents upstream food fermentation, in which fermenting organisms modify parent compounds during food production, delivering pre-transformed bioactives including isoflavone aglycones, minor ginsenosides, and pre-formed equol at ingestion. The lower track represents *in vivo* gut microbial biotransformation, in which ingested compounds are further converted in the colon by the resident microbiota. Both tracks converge at systemic bioavailability. The curved feedback arrow indicates that fermented food consumption also enriches resident biotransforming bacteria, amplifying *in vivo* conversion capacity.

A notable example of direct metabolite delivery is equol. Traditional Asian fermented soy pastes and aged preparations, such as miso and doenjang, can contain pre-formed equol generated by bacterial reductases during the fermentation process itself; this is less true of natto, whose dominant fermenting organism, *Bacillus subtilis*, hydrolyses isoflavone glycosides to aglycones but is not itself an equol-producing organism. Consuming equol directly through these aged, paste-type fermented soy products bypasses the need for colonic equol production entirely, which means that individuals who lack Slackia species in their colon may still derive the hormonal and cardiovascular benefits of this metabolite through such fermented food consumption (West et al., 2025). The fermentation organisms, pre-ingestion structural changes, gut microbiota effects, and downstream biotransformation impacts of the fermented foods discussed in this section are summarised in Table 3. The additive relationship between upstream food fermentation and *in vivo* gut microbial biotransformation, including the feedback loop through which fermented foods enrich the resident biotransforming microbiota, is depicted in Figure 3.

**TABLE 3:** Fermented foods, their principal fermentation organisms, the structural transformations undergone by bioactive compounds during fermentation prior to ingestion, reported effects on gut microbiota composition, and the downstream consequences for *in vivo* biotransformation capacity.

Fermented food	Key fermentation organism(s)	Bioactive compound(s) transformed	Structural change pre-ingestion	Effect on gut microbiota composition	Downstream biotransformation impact	References
Natto	*Bacillus subtilis* natto	Isoflavone glycosides	Glycoside to aglycone hydrolysis and poly-γ-glutamic acid generation	Promotes SCFA-producing taxa through cross-feeding mechanisms	Pre-transformed aglycones bypass initial absorption barriers to blunt early glycemic response	Cevallos-Fernández et al. (2026)
Tempeh	*Rhizopus spp.*, lactic acid bacteria	Isoflavone glycosides	Glycoside to aglycone hydrolysis	Enriches *Bifidobacterium* and *Akkermansia*	Combined delivery of aglycones and beneficial taxa optimizes systemic bioaccessibility	Cevallos-Fernández et al. (2026)
Miso	*Aspergillus oryzae*, lactic acid bacteria	Isoflavones, plant proteins	Glycoside to aglycone hydrolysis, proteolysis into ACE-inhibitory peptides	Promotes *Lactobacillus* expansion	Direct mucosal transport of pre-transformed peptides independently of colonic processing	Cevallos-Fernández et al. (2026)
Fermented Soymilk	*Lactiplantibacillus plantarum*, *Bifidobacterium breve*	Isoflavone glycosides (Daidzin)	Glycoside to aglycone hydrolysis (Daidzein)	Selectively enriches *Bifidobacterium* species	Expands endogenous β-glucosidase capacity to sustain secondary metabolite generation	Renukadevi et al. (2026)
Fermented Ginseng	Monascus ruber, *Lactobacillus spp.*	Major ginsenosides (Rb1)	Hydrolysis into minor ginsenosides (Rd, Rg3, Compound K)	Increases *Lactobacillus* abundance	Minor ginsenosides exhibit superior mucosal permeability and enhanced lipid regulation	Zhao et al. (2021)
Milk Kefir	*Lactobacillus kefiri, Kluyveromyces marxianus*	Milk proteins, lactose	Proteolytic cleavage to bioactive peptides, kefiran (EPS) synthesis	Enriches *Bifidobacterium* and *Lactobacillus*	Augments baseline enzymatic repertoire required for subsequent polyphenol deconjugation	Neves et al. (2026)
Water Kefir	*Lactobacillus paracasei,* *Saccharomyces*	Simple sugars	Synthesis of high-molecular-weight exopolysaccharides (dextran/levan)	Modulates endogenous EPS-consuming taxa	Establishes an auto-enrichment loop to stabilize the modified colonic microbial architecture	Yerovi-López and Gamero (2026)
Kimchi	*Leuconostoc mesenteroides, Lactiplantibacillus plantarum*	Glucosinolates, complex carbohydrates	Glucosinolate to isothiocyanate conversion, mannitol and GABA generation	Enriches *Akkermansia* muciniphila and Lachnospiraceae	Delivered isothiocyanates bypass host myrosinase requirement to directly activate antioxidant pathways	Cevallos-Fernández et al. (2026)
Sauerkraut	*Lactiplantibacillus plantarum, Leuconostoc spp.*	Glucosinolates	Glucosinolate to isothiocyanate conversion, organic acid accumulation	Shifts community toward SCFA-producing taxa	Directly delivers reactive secondary metabolites while extending bioaccessibility via acid modulation	Schropp et al. (2025)
Kombucha (green tea)	*Komagataeibacter*, *Saccharomyces* (SCOBY)	Gallated catechins	Tannase-mediated release of phenolic acids (gallic acid)	Decreases Bacillota to Bacteroidota ratio	Augmented systemic antioxidant currency and improved intestinal barrier integrity	Fraiz et al. (2024)
Pu-erh Tea	*Eurotium cristatum, Aspergillus niger*	Catechins	Oxidative polymerization to theabrownins, degallation	Modulates bile-acid metabolizing bacteria	Pre-transformed theabrownins enhance lipid metabolism and FXR signaling pathways	Cevallos-Fernández et al. (2026)
Sourdough Bread	*Lactiplantibacillus plantarum*, *Saccharomyces cerevisiae*	Cereal phenolic acids (chlorogenic acid)	De-esterification to caffeic and quinic acids, phytate degradation	Promotes colonic carbohydrate-fermenting taxa	Increased transepithelial diffusion kinetics of simplified phenolic derivatives	Cevallos-Fernández et al. (2026)
Yogurt	*Streptococcus* *thermophilus*, *Lactobacillus* *bulgaricus*	Milk proteins, glutamate	Proteolysis to bioactive peptides, GABA synthesis	Transient engraftment of LAB, enriches butyrate producers	Immediate enteric receptor signaling bypassing unpredictable colonic synthesis requirements	Cevallos-Fernández et al. (2026)
Fermented Milk (B. longum)	*Bifidobacterium longum* BB536	Tryptophan	Synthesis of indole-3-lactic acid (ILA)	Increases relative abundance of *Faecalibacterium*	Enhances immune signaling and mucosal tolerance via tryptophan metabolite salvage	Ejima et al. (2024)
Fermented Goji Juice	*Lactiplantibacillus plantarum, Lactococcus lactis*	Goji polysaccharides	Polysaccharide structural simplification	Elevates *Lactobacillus* abundance significantly	Restores gut microbiota homeostasis to actively inhibit uric acid production, mouse model; human translation not yet established	Ren et al. (2025)
Fermented Purslane	*Bacillus subtilis*, *Lactobacillus* reuteri	Flavonoid glycosides	Glycoside to aglycone hydrolysis, biogenic compound enrichment	Enhances *Faecalibacterium* prausnitzii abundance	Reinforces intestinal barrier tight junctions to optimize mucosal absorption kinetics	Cevallos-Fernández et al. (2026)
Fermented Chickpea	*Lactiplantibacillus plantarum*, *Weissella*	Tannins, glycosides	Tannase-driven depolymerization and phenol release	Enriches *Bifidobacterium* species	Reduces upper gastrointestinal transit barriers for low-molecular-weight phenolics	Sáez et al. (2022)

Pre-ingestion compound transformation and post-ingestion microbiota modulation are distinguished throughout as two separate and additive mechanisms. The Fermented Chickpea and Fermented Goji Juice rows have been revised per reviewer comment. Abbreviations: EPS, exopolysaccharide; GABA, gamma-aminobutyric acid; LAB, lactic acid bacteria; SCOBY, symbiotic culture of bacteria and yeast; SCFA, short-chain fatty acid.

## 5 Probiotics and prebiotics: targeted biotransformation enhancement

### 5.1 Probiotic strains as biotransformation agents

Probiotics offer a more targeted approach to altering biotransformation capacity than fermented foods, because defined strains can be selected for specific enzymatic properties. The key requirement is delivering live organisms that carry the genes for the relevant biotransformation enzymes, at a sufficient dose to produce measurable colonic activity (Rudzka et al., 2025).

Supplementation with *Lactiplantibacillus plantarum* WCFS1 introduces gallate decarboxylase and beta-glucosidase activity into the gut lumen, accelerating the conversion of polyphenol glycosides to aglycones (Shakya et al., 2021). WCFS1 is a well-characterised, extensively studied reference strain, but it is not itself a widely marketed commercial probiotic; most commercial *L. plantarum* products use different proprietary strains with enzyme profiles that have not necessarily been characterised to the same depth, a point of strain-level variability that is examined more fully in Section 5.4.

*Bifidobacterium* species carry broad-spectrum esterase and beta-glucosidase repertoires that improve the bioavailability of quercetin and isoflavone aglycones; the specific strains characterised by Peng et al. (2022) were B. adolescentis and *B. pseudocatenulatum*, although related esterase and beta-glucosidase activities have also been reported for *B. longum* and *B. animalis* strains in separate studies, and these activities should not be assumed to be uniform across the genus (Peng et al., 2022). Supplementation with *Lactobacillus* rhamnosus R0011 improved the bioavailability of glycyrrhizic acid from liquorice root in an animal model with liver injury (Li et al., 2022); because this finding derives from a rodent model, translation to humans should be treated cautiously given known species differences in gut microbiota composition between rats and humans, and direct human applicability has not yet been confirmed.

The most direct demonstration of phenotype conversion through probiotic supplementation comes from experiments using *Slackia equolifaciens* or *Slackia isoflavoniconvertens*. Introducing these organisms into the colon of equol non-producers provides the complete daidzein-to-equol enzymatic pathway that was previously absent (Gangwar et al., 2026; Renukadevi et al., 2026). It should be noted that the Gangwar et al. (2026) evidence for this is from an animal model (Albino Wistar rats) rather than humans, and the Renukadevi et al. (2026) reference is a review and engineering-focused discussion piece rather than a primary clinical trial of *Slackia* supplementation; direct human proof-of-concept evidence for phenotype correction via *Slackia* administration therefore remains limited, and this proof-of-concept should be understood as promising but not yet clinically established.

Dose, strain selection, and timing all influence whether probiotic supplementation produces measurable biotransformation changes. A minimum dose of approximately 1 × 10^10^ colony-forming units per day has been cited as a threshold for achieving consistent metabolic effects in human studies (Rudzka et al., 2025), although this threshold is a generalisation rather than a fixed rule: some well-characterised strains show measurable effects at lower doses around 10^9^ CFU/day, while others may require doses above 10^10^ CFU/day to achieve comparable effects, so dose requirements should be considered strain-specific rather than universal.

Whether to use single-strain or multi-strain preparations depends on the specific biotransformation pathway being targeted. Single strains allow clearer mechanistic attribution, while multi-strain products may support cross-feeding networks that sustain biotransformation activity over time, a possibility supported by the multi-species synbiotic trial of Napier et al. (2025) discussed in Section 5.3, though this rationale remains more readily demonstrated for synbiotic combinations specifically than for multi-strain probiotic products in general (Napier et al., 2025).

### 5.2 Prebiotic enrichment of biotransforming taxa

Prebiotics take a different approach: rather than introducing new organisms, they selectively enrich biotransforming bacteria that are already present in the colon. Inulin, fructooligosaccharides (FOS), and galactooligosaccharides (GOS) consistently support the expansion of *Bifidobacterium* and *Lactobacillus* populations, increasing the colonic pool of beta-glucosidase enzymes available for flavonoid deglycosylation (Pham et al., 2021; Holmes et al., 2022).

More targeted prebiotic approaches can enrich specific biotransforming taxa. Supplementation with pomegranate-derived polyphenol fractions provides selective substrate for *Eggerthellaceae*, enriching *Gordonibacter* species in the colon and increasing urolithin A production (Kuerec et al., 2024). This represents a polyphenol-mediated prebiotic effect, distinct from the classical fermentable fibre prebiotics such as inulin and FOS: under the broadened definition adopted by the International Scientific Association for Probiotics and Prebiotics (ISAPP), a substrate selectively utilised by host microorganisms conferring a health benefit, polyphenols that selectively enrich specific bacteria qualify as prebiotics in their own right, even though they act as a growth substrate for the specific bacteria that metabolise them rather than functioning as a broadly fermentable fibre that supports a wide range of saccharolytic taxa, as inulin or FOS do.

The critical variable is timing. Prebiotic supplementation needs to precede the target dietary bioactive by enough time to allow the relevant microbial populations to expand. Based on the available literature, a lead time of approximately two to 4 weeks is often sufficient to produce a detectable microbial population shift, though the precise duration required varies by prebiotic type and by baseline microbiota composition. If the polyphenol substrate arrives before the bacterial community has shifted, the biotransformation reaction proceeds at the pre-intervention rate (Holmes et al., 2022).

### 5.3 Synbiotic strategies

Synbiotics combine a specific probiotic organism with the prebiotic substrate that supports its activity. In the context of biotransformation, this means co-delivering a biotransforming bacterial strain alongside the dietary compound it metabolises. Administering Slackia species alongside daidzein-rich soy isoflavone extract provides both the enzymatic machinery and the substrate simultaneously, which in principle improves the likelihood of equol production regardless of the individual’s baseline phenotype (Renukadevi et al., 2026); in practice, this potential is constrained by the substantial technical challenge of delivering viable Slackia at doses sufficient to achieve durable colonic colonisation (see Section 5.4), so the approach should be understood as promising rather than as a guaranteed correction of non-producer status.

A multi-species synbiotic supplement containing several probiotic strains alongside polyphenol-rich pomegranate extract was shown in a randomised controlled trial to significantly increase urolithin A production and butyrate output while reducing inflammatory markers in healthy adults (Napier et al., 2025). The reported 49-fold increase in urinary urolithin A (Table 4) should be interpreted with some caution: a substantial part of this magnitude reflects very low or undetectable baseline urolithin A levels in some participants, including individuals who were effectively metabotype 0 at baseline, so the fold-change figure, while genuine, may overstate the absolute clinical impact relative to what it would suggest in a population with typical baseline urolithin A levels. The trial’s sample size (n = 32) is correspondingly modest and limits how confidently these findings generalise. This is nonetheless one of the few published trials to directly demonstrate that synbiotic supplementation can shift biotransformation phenotype in humans (Zolghadrpour et al., 2024).

**TABLE 4:** Published probiotic and prebiotic intervention studies reporting biotransformation or bioavailability outcomes for dietary bioactive compounds. Studies are arranged by substrate compound class and classified by design: randomised controlled trial (RCT), open-label human study, animal model, or *in vitro* co-culture.

Probiotic/prebiotic intervention	Study design	n	Duration	Compound/Substrate	Biotransformation outcome measured	Key finding	Limitation noted	References
Probiotics + soy isoflavones	RCT	NR	12 weeks	Soy isoflavones	Endocrine signalling and metabolic modulation	Improved reproductive hormones via modulated gut microbiota	Relies heavily on baseline community for terminal equol conversion; no direct urinary equol quantification reported	Renukadevi et al. (2026)
Synbiotic (prebiotic fiber) + isoflavones	RCT	NR	12 weeks	Daidzein/isoflavones	Lipid profile and BMI	Significant reduction in BMI and LDL cholesterol	Lacks specific metabolomic quantification of equol; benefit cannot be attributed to equol pathway specifically	Renukadevi et al. (2026)
*Enterococcus faecalis* MG3/MG4 + soy diet	[Animal] Rats	24	15 days	Daidzein	S-equol excretion	High concentration of S-equol generated (5.90–7.56 μg/g)	Small sample size; rat gut microbiota differs from human microbiota in key respects (e.g., higher native *Lactobacillus* abundance), limiting direct human applicability	Gangwar et al. (2026)
Engineered *Escherichia coli* Nissle 1917	[*In vitro*]	N/A	48 h	Daidzein	(S)-equol yield	High yield conversion achieved via optimized reductase activity	Strict regulatory hurdles limit immediate human translation; engineered strain may also compete poorly against established native microbiota *in vivo*	Renukadevi et al. (2026)
Multi-species synbiotic (DS-01) + pomegranate extract	RCT	32	91 days	Ellagitannins	Urinary Urolithin A	49-fold increase (p < 0.001) and 100 percent conversion of baseline non-producers to detectable urolithin A producers	Small sample size; large fold-change driven partly by very low/undetectable baseline levels in some participants	Napier et al. (2025)
Gordonibacter urolithinfaciens + ellagic acid	[Animal] Mice	NR	4 weeks	Ellagic acid	Urolithin bioavailability	Promoted Urolithin A production and expanded beneficial taxa	Minimal long-term intestinal colonization	Yuan et al. (2026)
*Bifidobacterium* pseudocatenulatum INIA P815	[*In vitro*]	N/A	48 h	Ellagic acid	Urolithin A generation	Produced 1.33 μM Urolithin A from 20 mg/L parent compound	Highly dependent on specific BHI culture medium	Yuan et al. (2026)
*Lactiplantibacillus* plantarum WCFS1	[*In vitro*]	N/A	24 h	Phenolic acids/flavonoids	Gallate decarboxylase activity	Specific generation of pyrogallol enhancing local bioavailability	Lacks *in vivo* pharmacokinetic validation	Shakya et al. (2021)
*Bifidobacterium* adolescentis + FOS/Inulin	[*In vitro*]	N/A	N/A	Complex flavonoid glycosides	Beta-glucosidase expression	Increased luminal pool of active flavonoid aglycones	Does not account for host phase II clearance mechanisms	Peng et al. (2022)
*Lactobacillus* acidophilus + prebiotics	RCT	NR	8 weeks	Plant glucosides	Circulating bioaccessible aglycones	Significantly expanded endogenous deglycosylation capacity	Evaluates 16S compositional data without direct enzyme assays	Li et al. (2022)
Targeted probiotic reductase augmentation	[Animal] Rats	NR	12 weeks	Berberine	Dihydroberberine (DHB) conversion	Approximately 5-fold increase in maximal plasma concentration (Cmax) via efflux evasion, as reported in the primary pharmacokinetic source	Rat model; the 5-fold figure is the source’s own headline (abstract) summary, but the underlying data show fold-differences ranging from approximately 3.25-fold–6.4-fold depending on the specific comparison (dhBBR- vs. BBR-treated animals, or antibiotic-depleted vs. conventional animals); direct human translation has not been established	Feng et al. (2015)
Gut microbiota-targeted dietary fiber + berberine	[Animal] Rats	24	8 weeks	Berberine	AMPK activation and fasting glucose	Enhanced DHB-producing taxa leading to superior glucose reduction	Animal model restricts direct human pharmacokinetic extrapolation	Seyed Hameed et al. (2020)
*Lactiplantibacillus* plantarum/kimchi matrix	RCT	NR	4 weeks	Glucosinolates	Isothiocyanate generation	Bypassed host myrosinase requirement resulting in rapid antioxidant response	Primarily driven by pre-transformation rather than *in vivo* activity	Cevallos-Fernández et al. (2026)
Precision prebiotics + glucoraphanin	RCT	NR	4 weeks	Glucoraphanin	Sulforaphane absorption and Nrf2 activation	High producers showed potent Nrf2 antioxidant pathway activation	Binary responder and non-responder divergence limits universal efficacy	Cevallos-Fernández et al. (2026)
Leuconostoc mesenteroides fermentation	[*In vitro*]	N/A	48 h	Glucosinolates	Thioglucosidase conversion	High yield conversion to highly reactive isothiocyanates	Lacks assessment of true systemic absorption kinetics	Cevallos-Fernández et al. (2026)

The key quantified finding and principal limitation are recorded for each study. The “Targeted Probiotic Reductase Augmentation” (berberine) row has been revised per reviewer comment to flag that the cited source is a review rather than a primary pharmacokinetic study. Abbreviations: AMPK, AMP-activated protein kinase; CFU, colony-forming units; Cmax, maximum plasma concentration; DHB, dihydroberberine; FOS, fructooligosaccharides; NR, not reported; RCT, randomised controlled trial.

Regulatory pathways for synbiotic products depend on jurisdiction. In the European Union, health claims for fermented or live biotherapeutic products require European Food Safety Authority evaluation, and novel microbial strains or compounds without a history of significant human consumption before 1997 may additionally fall under the EU Novel Food Regulation (Regulation (EU) 2015/2283), which requires a separate safety assessment before market authorisation. In the United States, products require generally recognised as safe (GRAS) determination or, for drug claims, an investigational new drug application. The specific pathway for products that claim to correct a biotransformation deficiency has not yet been formally established in either regulatory framework (Renukadevi et al., 2026).

### 5.4 Limitations and gaps in the evidence

Most probiotic biotransformation studies measure microbiota composition by 16S rRNA gene sequencing and use the resulting taxonomic data to infer changes in enzymatic activity. This inference is not always valid. Taxonomic abundance and enzymatic output are not the same thing, and gene expression data from metatranscriptomics, which measures which genes are actively being transcribed at the time of sampling, rather than which organisms or genes are merely present, are rarely included to confirm active enzyme production (Shahid, 2025).

Probiotic colonisation in the colon is largely transient. Most strains clear from the gastrointestinal tract within days to weeks of stopping supplementation, although persistence is itself strain-dependent: some strains, such as *Bifidobacterium longum* BB536, have been reported to persist for several weeks following supplementation, while others clear within days. In general, maintained biotransformation enhancement requires continuous dosing (Rudzka et al., 2025).

Enzymatic capacity is also strain-specific rather than species-wide, a point that deserves particular emphasis given how often species-level labelling is used in both the literature and on commercial products. Two isolates of the same *Lactobacillus* species can have very different beta-glucosidase profiles, strains of *Lactobacillus acidophilus*, for example, vary considerably in beta-glucosidase activity despite sharing a species designation, and commercial probiotic products often list species-level designations without identifying strains. This makes it difficult to determine which formulation provides a given biotransformation function (Napier et al., 2025). Published intervention studies examining the effect of probiotic and prebiotic supplementation on biotransformation capacity and bioavailability outcomes are compiled in Table 4.

### 5.5 Next-generation probiotics and prebiotics as nutraceuticals

Beyond their role in correcting specific biotransformation deficiencies, probiotics and prebiotics are increasingly positioned within a broader nutraceutical framework aimed at disease prevention rather than the correction of a single metabolic pathway. So-called next-generation probiotics, including genetically defined commensal strains selected or engineered for specific functional outputs, rather than conventional *Lactobacillus* and *Bifidobacterium* isolates chosen primarily for safety and ease of manufacture, are being investigated as nutraceutical agents intended to support metabolic and immune health more broadly, with disease prevention rather than treatment as the primary framing (Depommier et al., 2019; Wu et al., 2023). This shift in emphasis is conceptually consistent with the biotransformation phenotyping framework discussed in this review: identifying a person’s biotransformation phenotype could in principle help match next-generation probiotic or prebiotic nutraceuticals to the individuals most likely to benefit, although clinical evidence connecting phenotype status specifically to next-generation product selection remains at an early stage.

## 6 Health outcome divergence linked to biotransformation variability

Biotransformation phenotype differences translate directly into different health outcomes from identical dietary exposures. When clinical trials of dietary bioactives treat their study populations as homogeneous, the effects in producers and non-producers of the relevant metabolites cancel each other out, generating average results that may be misleading in both directions. Reviewing the evidence by phenotype reveals effects that mixed analyses obscure (Espín et al., 2024; Hu et al., 2024).

### 6.1 Cardiovascular and cardiometabolic outcomes

The cardiovascular benefit of soy isoflavones is largely restricted to equol producers. In a cross-sectional cohort analysis of healthy Japanese men, equol producers showed substantially lower rates of coronary artery calcification than non-producers consuming equivalent amounts of dietary isoflavones, with an odds ratio of 0.1 (95% CI: 0.01–0.90) (Ahuja et al., 2017); because this study was cross-sectional and conducted in a single-sex, single-population sample, it can establish association but not causation, and the direction and generalisability of the relationship should be interpreted with this design limitation in mind. Non-producers consuming the same diet show no significant improvement in endothelial function or blood pressure metrics. The cardiovascular protection attributed to soy in epidemiological studies from Asian populations, where equol production rates are higher, may be driven primarily by this subgroup.

Urolithin production phenotype similarly determines cardiometabolic responses to ellagitannin-rich foods. Overweight individuals classified as urolithin metabotype B show elevated baseline cardiometabolic risk markers compared to metabotype A subjects, and pomegranate extract interventions produce reductions in specific lipid parameters in metabotype B but not metabotype A individuals (Espín et al., 2024). The direction of causation underlying the metabotype B association is not established by this evidence: it is not yet clear whether the metabotype itself contributes mechanistically to the elevated risk markers, or whether some other factor associated with metabotype B status (for example, a shared dietary or metabolic influence on both the microbiota and cardiometabolic risk) accounts for both observations, and this uncertainty should be borne in mind when interpreting the metabotype B findings. Direct urolithin A supplementation, which bypasses the microbial conversion step, has been shown to reduce plasma C-reactive protein in randomised controlled trials and to decrease circulating acylcarnitines, indicating improved mitochondrial function (Liu et al., 2026).

Resveratrol response is highly variable across individuals and studies. As discussed in Section 2.1, host phase II metabolism (glucuronidation and sulfation via UGT and SULT enzymes) accounts for a substantial share of this variability independent of the microbiota, and microbial production of dihydroresveratrol is best understood as one contributing factor among several rather than the dominant explanation on its own. Individuals whose colonic bacteria reduce resveratrol to dihydroresveratrol without further degrading it to inactive products achieve higher plasma exposure to stilbene-derived metabolites and show greater cardiovascular and anti-inflammatory responses (Seyed Hameed et al., 2020). Urinary polyphenol excretion in the PREDIMED trial was associated with significantly lower cardiovascular disease risk, and part of this signal likely reflects inter-individual differences in microbial conversion efficiency (Domínguez-López et al., 2025).

### 6.2 Metabolic syndrome, obesity, and type 2 diabetes

Berberine’s glucose-lowering action depends on AMPK pathway activation, and its clinical effectiveness correlates with the microbial production of DHB, which determines how much of the alkaloid enters circulation. Individuals with high colonic reductase activity generate more DHB, escape P-glycoprotein efflux more completely, and show greater AMPK activation and fasting glucose reduction in diabetic populations (Seyed Hameed et al., 2020). It is worth noting that clinical trials of berberine typically use doses in the range of 1,500 mg per day, far exceeding the quantities available from dietary sources of berberine such as barberry or goldenseal; this gap between studied therapeutic doses and realistic dietary intake limits the direct translation of these findings into dietary advice, even though it remains directly relevant to supplement-based strategies discussed in Section 8.2, since berberine is also widely sold as an over-the-counter supplement in many countries at doses closer to those used in clinical trials, and the therapeutic-dose evidence is therefore not solely a dietary-source caveat.

As a separate line of evidence, one that does not itself demonstrate biotransformation of a dietary bioactive compound and is included here only for broader context, microbiome changes in patients with type 2 diabetes treated with metformin and liraglutide have been shown to correlate with metabolic outcomes (Glaros et al., 2025). This finding illustrates the general principle that the gut microbiota and pharmacological response are linked; it is not offered as direct support for the dietary biotransformation framework developed in this review and is presented here as an adjacent observation from the drug-microbiome literature rather than as integrated evidence for the biotransformation argument.

Quercetin supplementation produces measurable improvements in carbohydrate metabolism and insulin sensitivity in individuals whose microbiota can expand Bacteroidetes populations in response to the intervention, while those who lack this microbial response show no significant metabolic effect (Hu et al., 2024); this finding should be regarded as suggestive rather than definitively established, since the supporting citation addresses polyphenol-related gut metabotypes broadly rather than providing a study designed specifically to test this Bacteroidetes-response relationship.

The discrepancy in green tea catechin trial outcomes between Asian and Western cohorts in meta-analyses has been attributed in part to higher baseline abundance of biotransforming bacteria in Asian populations, resulting in more complete catechin deglycosylation and higher plasma metabolite concentrations (Espín et al., 2024); host phase II metabolism of catechins, which is itself subject to substantial inter-individual variation through functional polymorphisms in the UGT and SULT enzyme families (Scholl et al., 2018), also differs between populations and contributes independently to this discrepancy, so microbial biotransformation should be understood as a partial explanation rather than the complete account. It should be noted that the cited pharmacokinetic evidence for UGT/SULT polymorphism effects on catechin disposition derives from a European cohort characterising inter-individual rather than specifically Asian-versus-Western variation; a study directly comparing catechin-relevant UGT/SULT allele frequencies across these populations would strengthen this point further and is flagged here as a specific gap for future work.

Sulforaphane, derived from glucosinolates in cruciferous vegetables, activates the Nrf2 antioxidant pathway and has been associated with improved insulin sensitivity. High producers of sulforaphane, who have robust microbial thioglucosidase activity in addition to functional plant myrosinase, achieve Nrf2 activation at the same dietary intake levels that do not produce this effect in low producers (Cevallos-Fernández et al., 2026). A further, largely independent source of inter-individual variation arises after absorption: sulforaphane is a reactive electrophile that undergoes rapid glutathione conjugation, and variation in this host detoxification step adds a second layer of variability on top of differences in microbial production.

Separately from biotransformation phenotype specifically, probiotics have an independent evidence base in type 2 diabetes management. Probiotic supplementation has been associated with reductions in glycaemic and hyperlipidaemic markers in patients with type 2 diabetes mellitus, an effect attributed in part to decreased oxidative stress (Rittiphairoj et al., 2021; Baroni et al., 2024). Because obesity is itself a major risk factor for the development and progression of type 2 diabetes, the metabolic syndrome, obesity, and type 2 diabetes outcomes discussed in this section should be understood as interrelated through shared microbiota-linked mechanisms rather than as fully independent health domains.

### 6.3 Cancer risk reduction and anti-inflammatory outcomes

Soy consumption has shown a suggestive, though not uniformly confirmed, inverse association with breast cancer risk in prospective studies (Boutas et al., 2022), with this signal concentrated specifically in equol-producing women; the wider literature on soy and breast cancer risk is more mixed than this concentrated signal might suggest, and some studies have reported no protective effect even among equol producers, so the evidence should be characterised as suggestive rather than definitively protective. Equol binds oestrogen receptors with a profile that competes with endogenous oestrogen in a way that may reduce hormone-dependent cancer promotion. Non-producers show no significant cancer risk reduction at comparable soy intake levels, which may help explain why soy supplementation trials in unstratified Western populations have frequently reported null results (Espín et al., 2024).

Urolithin metabotype 0, affecting roughly 35% of adults, represents a complete inability to generate urolithins from dietary ellagitannins. Phase I and II clinical trials have established that individuals with this phenotype cannot activate mitophagy or reduce circulating inflammatory cytokines through intake of the specific ellagitannin-rich foods studied to date, principally pomegranate and walnut products, regardless of how much of these particular foods they consume (Kuerec et al., 2024); whether this finding generalises to all ellagitannin-rich foods, including less-studied sources, has not been directly established. Providing 1,000 mg of directly supplemented urolithin A to this population in randomised controlled trials produces a greater than six-fold increase in plasma urolithin A (p < 0.0001) and significant improvements in muscle endurance, including a substantially higher number of first dorsal interosseus muscle contractions until fatigue compared to placebo (Liu et al., 2022; Singh A. et al., 2022). Both of these urolithin A supplementation trials were sponsored by Amazentis, the manufacturer of the supplement under study, which is disclosed here in the interest of balanced appraisal and should be considered when weighing the strength of the evidence. This body of work nonetheless demonstrates that the biotransformation gap can be closed by formulation rather than dietary modification, when the microbial capacity is absent.

**FIGURE 4:**
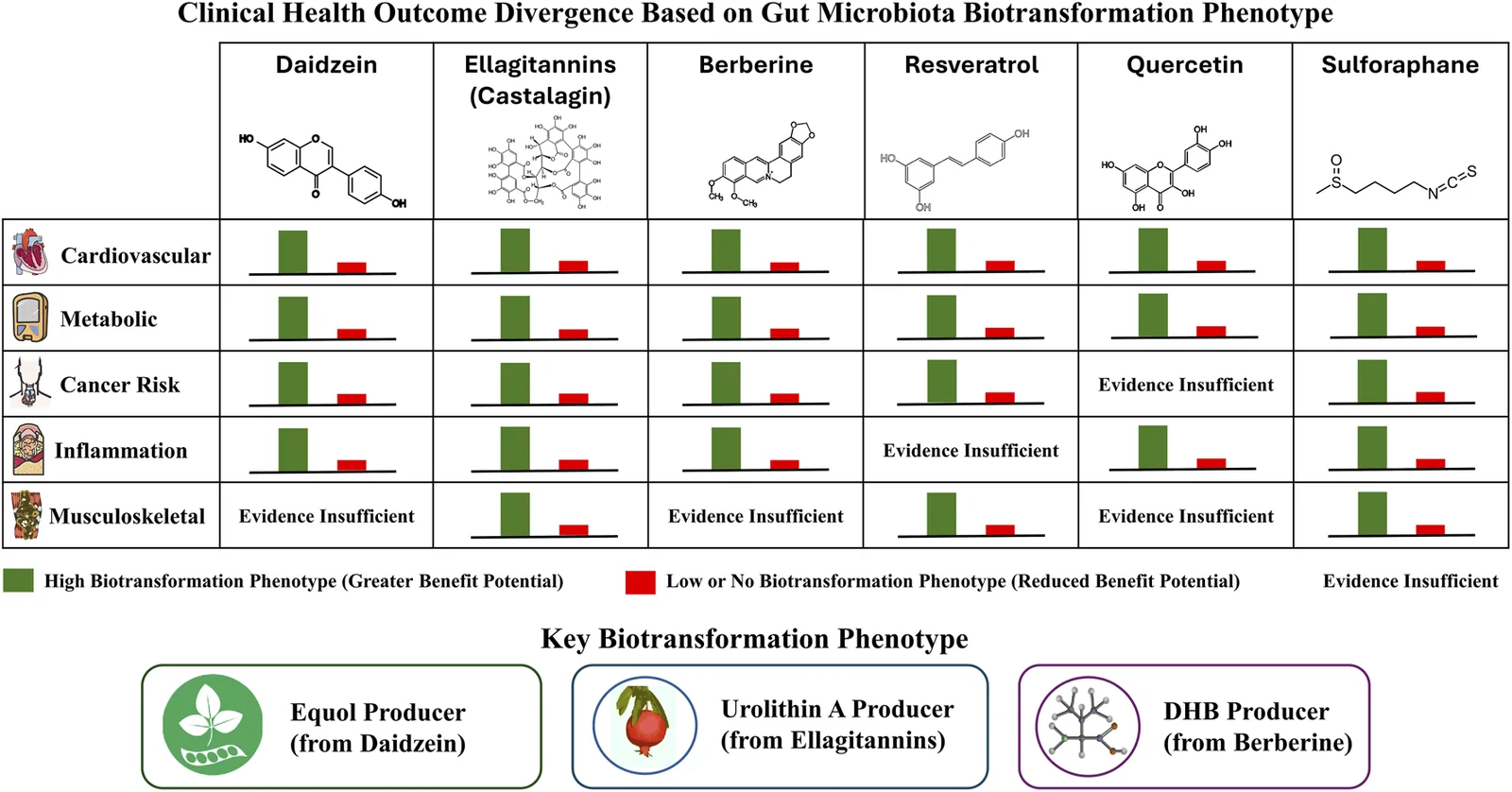
Clinical outcome divergence matrix showing differential health effects of six dietary bioactive compounds across five health domains, stratified by biotransformation phenotype. Paired bars in each cell represent benefit magnitude in individuals with high biotransformation capacity (green) and low or absent capacity (red), derived from published clinical data. Cells marked “Evidence Insufficient” indicate domains without phenotype-stratified data. Compounds: daidzein, ellagitannins, berberine, resveratrol, quercetin, sulforaphane. Phenotype icons below: soybean = equol producer; pomegranate = urolithin A metabotype; molecule = DHB producer. Abbreviations: DHB, dihydroberberine; Nrf2, nuclear factor erythroid 2-related factor 2. Approximate effect sizes for the key phenotype comparisons underlying this matrix are reported in the main text (Section 6), for example, an odds ratio of 0.1 (95% CI 0.01–0.90) for coronary artery calcification comparing equol producers to non-producers (Ahuja et al., 2017), and p < 0.0001 for the increase in plasma urolithin A following direct supplementation in metabotype 0 individuals (Singh A. et al., 2022).

Anthocyanin bioavailability and anti-inflammatory activity are determined by ring fission in the colon. The parent anthocyanin pigments are not detected at significant concentrations in plasma after consumption. The measurable systemic products are simple phenolic acids, including protocatechuic acid and vanillic acid, generated by specific ring-fission bacteria; it should be noted that these same phenolic acids also arise from the colonic breakdown of other polyphenol classes, including flavan-3-ols, so their presence in plasma cannot be attributed to anthocyanin metabolism with full specificity. The abundance of these organisms in an individual’s colon determines the plasma concentration of anti-inflammatory metabolites that circulates after an anthocyanin-rich meal (Di Pede et al., 2023). The divergence in health outcomes between individuals with high and low biotransformation capacity, across compound classes and health domains, is presented in Figure 4.

## 7 Methodological approaches to mapping biotransformation capacity

Defining an individual’s biotransformation phenotype requires methods that measure functional output rather than microbial community composition. Most studies to date have focused on taxonomy, establishing who is present in the gut microbiome. The clinically relevant question is what those organisms are producing from the compounds a person consumes (Shahid, 2025; Rengasamy, 2026).

### 7.1 Microbiome compositional profiling

16S rRNA amplicon sequencing is the most widely used method for gut microbiome analysis. It identifies microbial taxa by their ribosomal gene sequence and can detect the presence of established biotransforming organisms. An increase in Slackia abundance following an intervention, for example, provides indirect evidence of increased equol-producing capacity (Gangwar et al., 2026). The limitation is resolution: 16S sequencing identifies organisms at genus or species level under most standard analysis pipelines, and biotransformation capacity varies at the strain level within species. Amplicon sequence variant (ASV)-based approaches paired with higher-resolution reference databases can sometimes resolve sequences to species or, in favourable cases, near-strain level, but this remains the exception rather than routine practice in most biotransformation studies (Rudzka et al., 2025).

Shotgun metagenomic sequencing reads the full genomic content of microbial communities and allows direct detection of functional genes, including those encoding beta-glucosidases, beta-glucuronidases, ring-fission enzymes, and reductases. Mapping reads to databases such as KEGG and MetaCyc provides quantitative data on the genetic potential for specific biotransformation reactions (Tow et al., 2026), although this approach is constrained by the completeness of these reference databases: many biotransformation-relevant genes, particularly for less-studied compound classes, are not yet well annotated, so a negative result may reflect database gaps rather than a true absence of the corresponding enzymatic capacity. The further gap is that genetic potential is not the same as active enzyme expression. A gene can be present but not transcribed, and metatranscriptomic data are needed to confirm that relevant enzymes are being actively produced. Tools that impute function from 16S taxonomy, including PICRUSt2 and Tax4Fun2, are accessible and useful at the genus level but lose accuracy at the strain level, where biotransformation differences are most consequential (Schumacher et al., 2025); it should be noted that the Schumacher et al. (2025) validation was conducted in the context of a multiple sclerosis microbiome study rather than dietary biotransformation specifically, though the general point about strain-level imputation accuracy applies broadly across application domains (Schumacher et al., 2025).

### 7.2 Functional biotransformation assays

*Ex vivo* faecal fermentation is the most direct way to determine an individual’s biotransformation capacity without clinical intervention. A faecal sample provides a representative microbial community that is cultured under controlled anaerobic conditions with the target dietary compound as substrate. HPLC-tandem mass spectrometry (HPLC-MS/MS) then quantifies the parent compound and its metabolites over time, producing a conversion profile specific to that individual’s microbiota (Singh V. et al., 2022; Deyaert et al., 2023). This method is technically demanding in practice: it requires strict maintenance of anaerobic conditions throughout sample handling (Section 3.1), and results can be sensitive to the specific fermentation medium used and to the time elapsed between sample collection and the start of incubation, both of which are practical limitations for standardising the method across laboratories and for clinical implementation.

Sophisticated multi-compartment dynamic models, including the Simulator of the Human Intestinal Microbial Ecosystem (SHIME) and the TNO *in vitro* model of the colon (TIM-2), replicate the pH gradients, residence times, and sequential microbial compartments of the proximal, transverse, and distal colon (Zhu et al., 2024). These platforms allow the spatial and temporal kinetics of biotransformation to be mapped with a degree of detail that batch fermentation cannot provide, although the specialised equipment and operational expertise they require carry a substantial cost that limits their availability, and validation of their output against true *in vivo* metabolite profiles is not yet complete for all compound classes. They have been used to assess how transit time affects the extent of polyphenol conversion and how community changes alter metabolite output (Minnebo et al., 2023).

In clinical settings, biotransformation phenotyping can be performed non-invasively through urine or plasma metabolomics following a standardised dietary loading dose. Administering a controlled quantity of ellagitannins or soy isoflavones and measuring urolithin and equol concentrations in urine over the following 24–48 h categorises individuals into producer and non-producer metabotypes with good reproducibility for clearly positive or clearly negative individuals (Espín et al., 2024; Jarrín-Orozco et al., 2025); reproducibility is comparatively lower for borderline producers, individuals who generate small but detectable amounts of the target metabolite, and classification of this group is more sensitive to assay timing and threshold choice.

Faecal beta-glucuronidase activity assays provide a direct measure of the colonic capacity for deconjugating phase II metabolites, quantifying one component of enterohepatic recycling efficiency with a straightforward enzymatic assay on a faecal sample (Deng et al., 2026); because enterohepatic recycling depends on a broader metabolic network than beta-glucuronidase activity alone, this assay should be understood as informative about one specific step in the process rather than as a complete characterisation of recycling efficiency.

### 7.3 Computational prediction tools

BioTransformer 3.0 is a knowledge-based prediction tool, incorporating some machine learning elements, that predicts the gut microbial metabolites likely to be produced from a given parent compound, taking the molecular structure as input (Wishart et al., 2022). It provides a rapid initial screen for likely biotransformation products and has been validated against experimentally confirmed metabolites for a substantial number of polyphenols and alkaloids. Because the tool predicts metabolites based on a defined library of known reaction rules, it may not anticipate genuinely novel biotransformation pathways that fall outside this rule set; consistent with this, its accuracy is highest for well-characterised substrate classes and lower for novel or complex structures.

Genome-scale metabolic models (GEMs) of gut microbial communities, combined with flux balance analysis (FBA), allow *in silico* simulation of metabolite fluxes through defined microbial networks under specified nutrient conditions (Ruiz-Moreno et al., 2025). These models can predict which bacterial species will be metabolically active when a specific dietary compound is available as a substrate and can identify potential cross-feeding interactions that support sequential biotransformation reactions. Their practical use is currently limited by substantial computational cost and by the need for detailed input data, including individual microbial abundance profiles, nutrient availability, and kinetic parameters, much of which is not routinely available. Integrating metagenomic data from an individual into these models offers a route to personalised biotransformation prediction, though the computational requirements and data quality needs remain substantial.

More broadly, the practical implementation of personalised nutrition based on biotransformation phenotype is expected to depend on the continued maturation of several adjacent technical fields, rather than on phenotyping methods alone. These include large-scale metagenomic profiling of individuals, the application of machine learning approaches to integrate multi-omic data and predict biotransformation phenotype from more easily obtained inputs, the falling cost and increasing throughput of next-generation sequencing, and the growth of population-level microbiome reference databases against which an individual’s profile can be compared (Schirmer et al., 2016; Al Bander et al., 2020). The methodological tools available for mapping individual biotransformation capacity, including their resolution, cost, relevance, and key limitations, are compared in Table 5.

**TABLE 5:** Comparative overview of methodological tools for characterising gut microbiota biotransformation capacity.

Method	What it measures	Resolution	Cost	Biotransformation relevance	Key limitation	References
16S rRNA amplicon sequencing	Taxonomic composition and relative abundance of the microbial community	Genus-level identification	Low analytical cost permitting large-scale population screening	Identifies the baseline presence of potential biotransforming taxa such as equol-producing Slackia	Taxonomic presence does not confirm functional capacity or active enzymatic output (composition vs. function gap)	Rudzka et al. (2025)
Shotgun metagenomics	Microbial functional gene potential and comprehensive taxonomic composition	Strain-level taxonomy and functional gene-level mapping	Moderate to high cost driven by required sequencing depth and intensive computational resources	Maps the specific genetic coding for hydrolytic and reductive enzymes within the colonic ecosystem	Gene presence does not guarantee active expression, and reference-database annotation of biotransformation genes remains incomplete	Ruiz-Moreno et al. (2025)
Metatranscriptomics	Actively expressed microbial genes via RNA transcripts	Gene-expression and transcript-level profiling	High cost due to RNA instability, extraction complexity, and required sequencing depth	Identifies actively transcribed biotransformation pathways responding directly to dietary bioactive exposure	Transcript abundance does not always correlate with final translated protein levels or measured metabolite output	Shahid (2025)
*Ex vivo* faecal fermentation (SHIME, TIM-2, batch)	Dynamic microbial processing and real-time secondary metabolite generation	Highly resolved functional metabolite and community-level tracking	Extremely high equipment and operational costs for dynamic, multi-compartment platforms	Serves as the functional gold standard for mapping individual parent-to-metabolite degradation cascades	Excludes host variables including intestinal transport, phase II metabolism, and enterohepatic recycling, and results are sensitive to medium choice and sample handling time	Zhu et al. (2024)
Urine metabolomics	Excreted bioavailable secondary metabolites following parent compound ingestion	Precise metabolite-level functional output	Moderate to high cost linked to high-resolution mass spectrometry and authentic analytical standards	Non-invasive phenotyping tool that stratifies human cohorts into producer and non-producer metabotypes with good reproducibility for clear responders	Difficult to attribute the exact microbial origin of a given metabolite without paired metagenomics, and reproducibility is lower for borderline/low producers	Shahid (2025)
Faecal enzyme activity assay	Specific catalytic output of the luminal microbial community	Targeted enzyme-level functional activity	Low to moderate cost utilizing standard colorimetric or fluorometric substrates	Accurately quantifies specific hydrolytic capabilities such as beta-glucuronidase-driven enterohepatic recycling	Captures only a single enzyme class and does not characterise the broader interconnected metabolic network	Deng et al. (2026)
BioTransformer 3.0	Predicted metabolic transformation products generated by the gut microbiota	Specific structural-level metabolite prediction from input SMILES strings	Free or negligible computational cost utilizing an open-access web server	Accelerates hypothesis generation by simulating expected secondary metabolites derived from complex dietary precursors	Predictions are bounded by a defined library of known reaction rules and cannot be expected to anticipate genuinely novel pathways	Wishart et al. (2022)
PICRUSt2/Tax4Fun2	Imputed functional genomic profiles derived from 16S rRNA taxonomic data	Imputed gene and pathway-level mapping based on reference genomes	Low computational cost leveraging existing 16S rRNA amplicon datasets	Provides a baseline estimation of the total enzymatic repertoire present within the host ecosystem	Predictive accuracy degrades sharply at the strain level due to horizontal gene transfer among closely related taxa	Douglas et al. (2020)
Gut-on-a-chip models	Spatial and temporal kinetics of microbial biotransformation combined with epithelial transport	System-level modelling of the host-microbiome interface	Very high cost driven by microfluidic device fabrication and complex co-culture maintenance	Accurately replicates region-specific pH gradients, cellular interactions, and metabolite mucosal permeability	Technically demanding to sustain the obligate anaerobic-aerobic interface required for true colonic simulation, limiting throughput	Zhu et al. (2024)
Clinical microbiome panel tests	Targeted taxonomic or functional microbial biomarkers from clinical biospecimens	Varies from genus-level presence to targeted functional gene detection	Moderate analytical cost designed specifically for scalable clinical implementation	Facilitates practical translational guidance by matching targeted dietary interventions to verified individual metabolic capabilities	Restricted by a regulatory vacuum regarding standardized biomarker thresholds for actionable health claims, and no specific commercial products are evaluated here	Shahid (2025)

For each method, the measured output, functional resolution, approximate relative cost, relevance to biotransformation phenotyping, and principal limitation are described. Methods range from population-level compositional screening to precision functional platforms and computational prediction tools. Abbreviations: FBA, flux balance analysis; GEM, genome-scale metabolic model; HPLC-MS/MS, high-performance liquid chromatography with tandem mass spectrometry; SHIME, Simulator of the Human Intestinal Microbial Ecosystem; SMILES, simplified molecular-input line-entry system; TIM-2, TNO *in vitro* model of the colon.

## 8 Personalised nutrition: translational implications and future directions

### 8.1 From population recommendations to phenotype-stratified guidance

Current dietary guidelines recommend polyphenol-rich foods for the general population based on population-level risk reductions observed in epidemiological studies. Specific polyphenol-related guidance is issued primarily by national and regional authorities, for example the United States Dietary Guidelines for Americans, the United Kingdom’s Eatwell Guide, and Mediterranean diet guidance promoted across southern European countries, rather than by the Food and Agriculture Organization or World Health Organization directly, whose broader guidance on fruit and vegetable intake is not specific to polyphenols. The biotransformation evidence reviewed here shows that these population-level benefits are distributed unevenly. For equol-dependent outcomes such as menopausal symptom relief and cardiovascular protection from soy, the benefit accrues to producers and not to non-producers consuming the same diet (Jarrín-Orozco et al., 2025).

In a randomised crossover trial in postmenopausal women, improvements in vasomotor symptoms and quality of life following consumption of red clover and soy isoflavones occurred exclusively in women with the microbial machinery to produce equol. Non-producers consuming identical preparations showed no significant change in symptom scores or quality of life measures (Jarrín-Orozco et al., 2025). Because this trial was conducted specifically in postmenopausal women, its findings may not generalise without further study to perimenopausal women or to women with differing hormonal profiles, for whom the relationship between equol status and symptom response has not been established with the same evidence base. This is nonetheless a clinical demonstration that unstratified dietary recommendations can be simultaneously correct at the population level and irrelevant at the individual level.

The microbiota-determined biotransformation capacity functions as a nutritional analogue of pharmacogenomics. Hepatic cytochrome P450 variants such as CYP2D6 and CYP2C19 are now routinely tested before prescribing certain drugs, because these variants determine how quickly the drug is metabolised and whether therapeutic concentrations are reached, although it is worth noting that even CYP450 genotyping, despite its longer history and clearer regulatory pathway, is not yet implemented routinely in all clinical settings; similar or greater implementation challenges should be anticipated for biotransformation phenotyping given its comparatively earlier stage of development. The colonic enzymatic repertoire plays a structurally similar role for dietary bioactives: it determines the conversion rate from inactive precursor to active metabolite (Espín et al., 2024).

An individual consuming a mixed polyphenol diet may simultaneously be a urolithin A producer, an equol non-producer, and a dihydroresveratrol producer, creating a composite biotransformation profile with distinct implications for which dietary compounds will produce systemic effects in that person (Hu et al., 2024). As a concrete illustration, such an individual would be expected to derive the cardioprotective and mitophagy-related benefits of pomegranate or walnut consumption in full, to derive negligible hormonal or cardiovascular benefit from dietary soy isoflavones unless equol itself or an equol-containing fermented soy product were supplied directly, and to retain at least partial benefit from resveratrol-containing foods via the dihydroresveratrol pathway; this single hypothetical case underscores why a single dietary recommendation cannot be expected to serve all three pathways equally well for the same person. Treating equol production (present in roughly 30%–50% of adults, depending on population), urolithin A metabotype (roughly 65% of adults, i.e., excluding the approximately 35% who are metabotype 0), and dihydroresveratrol production as approximately independent traits for the purpose of illustration only, the specific three-way combination described above would be expected in a minority of individuals, on the rough order of one in five to one in ten adults, depending on population-specific producer frequencies, which helps convey why phenotype-guided recommendations cannot be reduced to a single dominant subgroup. This figure is an illustrative back-of-envelope estimate under a simplifying independence assumption rather than an empirically measured joint frequency, since the three traits have not, to the authors’ knowledge, been assessed jointly in a single cohort.

### 8.2 Phenotype-guided intervention design

Identifying a non-producer phenotype shifts the intervention logic from dietary modification to metabolic bypass or microbiota correction. Three strategies are available. The first is direct supplementation with the biotransformation product rather than the dietary precursor. Urolithin A is commercially available as a supplement and has been tested in Phase I and II clinical trials, where it produces consistent systemic exposure levels across the full population, including metabotype 0 individuals who cannot generate it from dietary sources (Liu et al., 2022; Singh A. et al., 2022; Kuerec et al., 2024); as noted in Section 6.3, these supporting trials were industry-sponsored, and urolithin A is marketed and regulated as a dietary supplement rather than as a drug, meaning it has not been subject to the same rigorous, independently-replicated clinical testing requirements that would apply to a pharmaceutical product. Dihydroberberine supplements follow the same logic for berberine non-responders, and in some jurisdictions dihydroberberine may be classified as a novel food ingredient subject to additional regulatory review before it can be marketed. The second strategy is targeted probiotic supplementation to introduce the missing biotransforming organism, as discussed in Section 5. The third is the use of fermented food products that already contain the biotransformed metabolite, such as equol-containing fermented soy preparations for equol non-producers (West et al., 2025); of the three strategies, this third one is the least supported by direct evidence, since the concentration of pre-formed metabolites such as equol in fermented foods is often low and variable between products and batches, and this should be regarded as the weakest of the three options for non-producers who need a reliable and quantifiable dose.

### 8.3 Future research priorities

The most pressing methodological need is standardised phenotyping protocols. Currently, studies use different polyphenol loading doses, different urine collection windows, different analytical methods, and different threshold values for defining producer versus non-producer status. At minimum, standardisation would need to specify: the loading dose for each target compound (for example, a defined quantity of pomegranate extract for ellagitannin phenotyping, or a defined quantity of soy isoflavone extract for equol phenotyping); the sample collection window following the loading dose (commonly proposed as 24 versus 48 h, though consensus is lacking); the analytical concentration threshold that defines producer versus non-producer status for each metabolite; and the degree of background dietary standardisation required during the testing period to avoid confounding from habitual intake. Without harmonised protocols along these lines, results cannot be directly compared across studies, and the field cannot converge on clinical decision thresholds (Espín et al., 2024; Zhu et al., 2024).

A second priority is the re-analysis of existing randomised controlled trial datasets with biotransformation phenotype as a pre-specified stratification variable. Many polyphenol intervention trials collected baseline stool or urine samples that could be used retrospectively to classify participants as producers or non-producers; large dietary trials that collected baseline biospecimens suitable for phenotyping, alongside the various published soy isoflavone intervention trials discussed throughout this review, represent plausible candidates for this kind of retrospective stratification. PREDIMED itself collected urinary polyphenol data (Domínguez-López et al., 2025), but, to the authors’ knowledge, did not systematically collect stool samples for microbiome analysis; retrospective phenotype stratification by stool metagenomics would therefore not be possible for this trial specifically, and it is referenced here as an example of the biospecimen-driven reasoning that should guide candidate selection for re-analysis, rather than as a trial with confirmed microbiome-ready samples. Re-analysing such datasets by phenotype could rescue apparent null results and confirm or refute whether reported positive effects were phenotype-dependent (Hu et al., 2024).

Longitudinal data on the stability of biotransformation phenotypes over time are lacking. Cross-sectional studies and short-term interventions dominate the literature, but the relevant clinical question is whether an individual’s equol or urolithin producing status changes over decades as their diet, age, and microbiota composition evolve, and whether targeted interventions can produce durable phenotype shifts rather than transient ones. Large-scale longitudinal cohort efforts such as the ZOE PREDICT studies have begun to generate the kind of repeated-measures, individual-level metabolic data that this question requires, although these efforts are recent, were not designed specifically around biotransformation phenotyping, and the relevant data are still emerging (Rengasamy, 2026).

Advanced modelling platforms, including gut-on-a-chip systems and multi-compartment fermentation models, offer the possibility of using an individual’s microbial community to predict biotransformation product profiles *in vitro* before designing dietary or supplementation strategies (Zhu et al., 2024; Ruiz-Moreno et al., 2025). Implementing this as a clinical tool will require substantial standardisation, including agreement on which reference microbial community or communities should be modelled, which culture medium and nutrient conditions should be used, and which specific metabolites and quantification methods should constitute the standard analytical readout; each of these choices currently varies between research groups and remains an ongoing challenge for the field.

**FIGURE 5:**
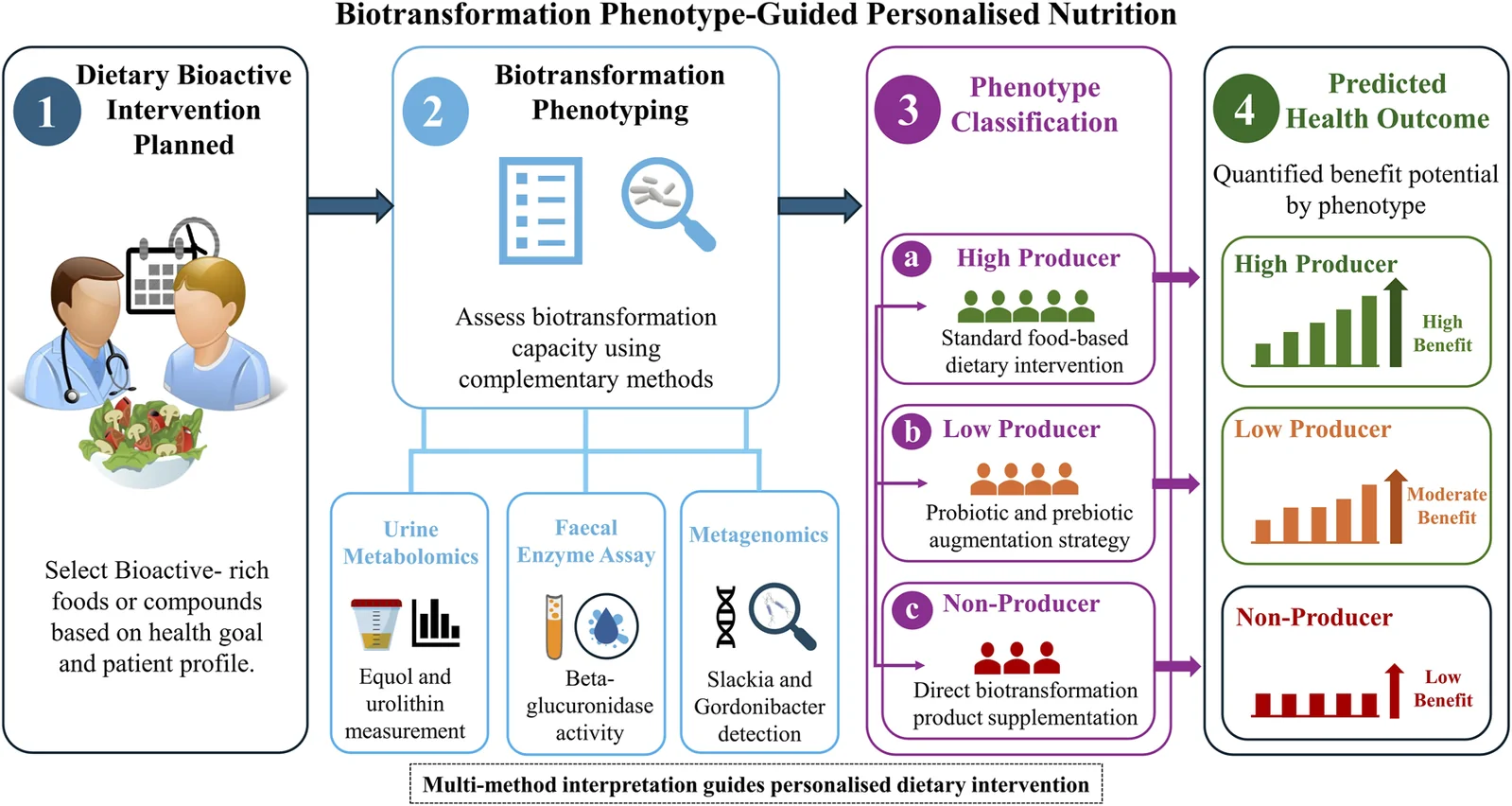
Proposed clinical workflow for biotransformation phenotype-guided personalised nutrition. Stage 1: dietary bioactive intervention is planned. Stage 2: phenotyping is performed by urine metabolomics, faecal beta-glucuronidase activity assay, or gut metagenomics. Stage 3: individuals are classified as high producer, low producer, or non-producer. Stage 4: intervention is matched to phenotype, standard dietary advice for high producers, probiotic or prebiotic augmentation for low producers, and direct biotransformation product supplementation for non-producers. Abbreviations: CFU, colony-forming units; GRAS, generally recognised as safe; UA, urolithin A. The choice among these three phenotyping methods depends on the target compound and clinical context. Urine metabolomics is best suited to compound-specific phenotyping such as urolithin or equol producer status; faecal beta-glucuronidase activity assay measures a single enzymatic step relevant chiefly to enterohepatic recycling; and gut metagenomics is most informative for characterising baseline microbiota composition rather than a single metabolite’s producer status.

A regulatory gap exists at the intersection of microbiome science and nutrition policy. The US Food and Drug Administration, together with the International Council for Harmonisation, has begun modernising its framework for evaluating drug-drug interactions, for instance through the “M12 Drug Interaction Studies” guidance (FDA/ICH, Docket No. FDA-2022-D-1527; draft issued August 2022, finalised August 2024), which updates how enzyme- and transporter-mediated pharmacokinetic interactions are assessed; however, this guidance is centred on drug-drug interactions rather than on the gut microbiome specifically, and does not address gastrointestinal or microbiome-mediated drug interactions to the same extent as some existing regional guidance. More microbiome-specific regulatory recognition remains limited and fragmented, and, to the authors’ knowledge, no FDA or European Food Safety Authority framework yet formally addresses the gut microbiome’s role in dietary bioactive metabolism specifically. Neither the FDA nor the European Food Safety Authority has established a formal framework for evaluating health claims based on biotransformation phenotype stratification. Developing such a framework is a precondition for approving personalised dietary guidance tools and phenotype-matched functional food products (Renukadevi et al., 2026). A proposed clinical workflow for implementing biotransformation phenotype-guided personalised nutrition, from initial phenotyping through to intervention selection and predicted outcome, is illustrated in Figure 5.

A mechanistic thread that runs through several of the health outcomes discussed in this review, and that merits more explicit treatment, is the role of gut microbial metabolites in immune modulation. Beyond the specific biotransformation products discussed above (equol, urolithins, dihydroberberine), gut microbial metabolites more broadly, including short-chain fatty acids and tryptophan-derived indoles, act on host immune signalling pathways and contribute to the resolution of inflammation, providing a plausible shared mechanistic basis for several of the anti-inflammatory associations described in Section 6 (Wang et al., 2023; Vargas et al., 2025). A fuller mechanistic account of these immunomodulatory pathways is available in the specialised literature on gut microbial metabolites and immune function; readers seeking this level of detail are referred there, since a complete treatment of immune signalling mechanisms falls outside the scope of a review focused specifically on dietary bioactive biotransformation.

## 9 Conclusion

The health benefit derived from a dietary bioactive compound is not determined by intake alone. Dietary intake remains a necessary precondition, without consumption of the parent compound, no metabolite can be produced, but given adequate intake, the health benefit realised is determined by how much of the active metabolite reaches systemic circulation, and that quantity depends on the enzymatic capacity of the individual’s gut microbiota. This review has organised the determinants of that capacity into three factors: baseline microbiota composition shaped by long-term diet, age, and geographic background; fermented food consumption that delivers pre-transformed compounds and modifies the microbial community; and targeted probiotic and prebiotic interventions that enrich specific biotransforming populations. Together, these three factors define an individual’s biotransformation phenotype.

These three factors should not be understood as fully independent: fermented food consumption sustained over years can itself reshape baseline microbiota composition, and probiotic or prebiotic supplementation can be deployed specifically to correct deficiencies in an individual’s baseline phenotype, so the framework is best understood as three interacting levers acting on a single underlying biotransformation capacity rather than three separate, additive inputs.

The clinical implications of this phenotype are not trivial. Equol production determines the cardiovascular and hormonal response to soy isoflavones, and an estimated 50%–70% of Western adults and a smaller proportion of Asian adults are non-producers who would not be expected to benefit from this pathway. Urolithin metabotype determines whether ellagitannin-rich foods activate mitophagy and reduce inflammation, and roughly 35% of adults fall into metabotype 0, unable to generate any urolithins from dietary ellagitannins. Dihydroberberine production determines the glucose-lowering efficacy of berberine in metabolic disease. In each case, individuals with the same dietary intake but different biotransformation phenotypes show measurably different health trajectories. Treating these populations as equivalent in clinical trials produces misleading average results and has contributed to the inconsistency that characterises the polyphenol intervention literature.

Three gaps need to be closed to translate this understanding into clinical practice. First, standardised phenotyping protocols are needed. The loading dose, sample collection window, and analytical thresholds for classifying equol and urolithin producer status must be harmonised internationally before phenotype-stratified trial designs and clinical phenotyping tests can be implemented reliably. Second, existing randomised controlled trial datasets should be re-analysed with biotransformation phenotype as a stratification variable. This represents a low-cost route to generating phenotype-stratified evidence from already-collected data. Third, regulatory frameworks for biotransformation-phenotype-based health claims require formal development. The tools to carry out phenotyping in clinical practice already exist, in the form of urine metabolomics, faecal enzyme assays, and metagenomics. The framework to act on the results does not.

Moving dietary bioactive research toward phenotype-guided personalised nutrition requires no fundamentally new biotechnology. It requires agreement on methods, willingness to re-examine existing data through a new analytical lens, and regulatory structures that recognise microbial metabolic capacity as a clinically meaningful biological variable. It is worth being explicit, however, that the existing tools, urine metabolomics, faecal enzyme assays, and metagenomics, are not yet widely available in routine clinical practice, and translating this conceptual readiness into practical implementation will additionally require investment in clinical infrastructure, laboratory capacity, and clinician training, alongside the methodological and regulatory developments described above. Point-of-care tests for equol and urolithin phenotypes are under active development and may become more widely available in the coming years, which would help address this limitation, though such tests have not yet reached routine clinical deployment.
